# Transcription analysis of neonicotinoid resistance in Mediterranean (MED) populations of *B. tabaci* reveal novel cytochrome P450s, but no nAChR mutations associated with the phenotype

**DOI:** 10.1186/s12864-015-2161-5

**Published:** 2015-11-14

**Authors:** Aris Ilias, Jacques Lagnel, Despoina E. Kapantaidaki, Emmanouil Roditakis, Costas S. Tsigenopoulos, John Vontas, Anastasia Tsagkarakou

**Affiliations:** Hellenic Agricultural Organisation - “DΕMETER”, NAGREF - Institute of Olive Tree, Subtropical Crops and Viticulture, Heraklion, Greece; Institute of Marine Biology, Biotechnology and Aquaculture (IMBBC), Hellenic Centre for Marine Research (HCMR), Heraklion, Greece; Department of Environmental and Natural Resources, University of Patras, Agrinio, Greece; Department of Crop Science, Agricultural University of Athens, Athens, Greece; Institute of Molecular Biology and Biotechnology, Foundation of Research and Technology, Heraklion, Greece

**Keywords:** *Bemisia tabaci*, RNA-Seq, Neonicotinoids, Resistance mechanisms

## Abstract

**Background:**

*Bemisia tabaci* is one of the most damaging agricultural pests world-wide. Although its control is based on insecticides, *B. tabaci* has developed resistance against almost all classes of insecticides, including neonicotinoids.

**Results:**

We employed an RNA-seq approach to generate genome wide expression data and identify genes associated with neonicotinoid resistance in Mediterranean (MED) *B. tabaci* (Q1 biotype). Twelve libraries from insecticide resistant and susceptible whitefly populations were sequenced on an Illumina Next-generation sequencing platform, and genomic sequence information of approximately 73 Gbp was generated.

A reference transcriptome was built by *de novo* assembly and functionally annotated. A total of 146 P450s, 18 GSTs and 23 CCEs enzymes (unigenes) potentially involved in the detoxification of xenobiotics were identified, along with 78 contigs encoding putative target proteins of six different insecticide classes. Ten unigenes encoding nicotinic Acetylcholine Receptors (nAChR), the target of neoinicotinoids, were identified and phylogenetically classified. No nAChR polymorphism potentially related with the resistant phenotypes, was observed among the studied strains.

DE analysis revealed that among the 550 differentially (logFC > 1) over-transcribed unigenes, 52 detoxification enzymes were over expressed including unigenes with orthologues in P450s, GSTs, CCE and UDP-glucuronosyltransferases.

Eight P450 unigenes belonging to clades CYP2, CYP3 and CYP4 were highly up-regulated (logFC > 2) including CYP6CM1, a gene already known to confer imidacloprid resistance in *B. tabaci*. Using quantitative qPCRs, a larger screening of field MED *B. tabaci* from Crete with known neonicotinoid phenotype was performed to associate expression levels of P450s with resistance levels. Expression levels of five P450s, including CYP6CM1, were found associated with neonicotinoid resistance. However, a significant correlation was found only in CYP303 and CYP6CX3, with imidacloprid and acetamiprid respectively*.*

**Conclusion:**

Our work has generated new toxicological data and genomic resources which will significantly enrich the available dataset and substantially facilitate the molecular studies in MED *B. tabaci*. No evidence of target site neonicotinoid resistance has been found. Eight P450 unigenes, including CYP6CM1, were found significantly over-expressed in resistant *B. tabaci*. This study suggests at least two novel P450s (CYP303 and CYP6CX3) as candidates for their functional characterization as detoxification mechanisms of neonicotinoid resistance in *B. tabaci.*

**Electronic supplementary material:**

The online version of this article (doi:10.1186/s12864-015-2161-5) contains supplementary material, which is available to authorized users.

## Background

The whitefly *Bemisia tabaci* (Hemiptera: Aleyrodidae) (sweet potato or tobacco whitefly) is a broad phloem feeding herbivore. *B. tabaci* is a complex of 31 putative morphocryptic species [1] and one of the most damaging pests of protected, field crops and ornamentals worldwide [2]. The current global status of *B. tabaci* as a pest refers mainly to two species of the complex, MEAM1 (Middle East-Asia Minor I, formerly referred to as biotype B) and MED (Mediterranean, formerly referred to as biotype Q) which are both highly invasive and polyphagous (over 1000 host plants reported) and particularly detrimental by transmitting viruses which cause serious crop diseases.

Currently, the control of *B. tabaci* relies on insecticides. However, their extensive and chronic use has imposed strong selection pressures for resistance to many insecticide classes in *B. tabaci*, including neonicotinoids, which are the world market leaders of insecticides [3–7]. Neonicotinoids act by binding on insect nicotinic acetylcholine receptors (nAChR). Imidacloprid was the first neonicotinoid introduced in the market in 1991 followed by acetamiprid, thiamethoxam, thiacloprid and clothianidin [8].

Insecticide resistance is typically characterized by a variety of molecular aberrations, such as transcriptional changes, gene amplification and point mutations in coding regions, which in turn result in phenotypic novelties like increased rates of insecticide detoxification or reduced sensitivity of the target protein(s). The most significant mechanisms of resistance to neonicotinoid insecticides are decreased target-site sensitivity and increased metabolic detoxification by cytochrome P450 monooxygenages [9, 10]. In *B. tabaci*, it was shown that the cytochome P450 CYP6CM1 is the major resistance mechanism, as the gene expression is highly associated with the resistance phenotype in a number of populations worldwide [11], and it encodes a protein capable of metabolising neonicotinoids (such as imidacloprid and thiacloprid) to less toxic metabolites [10].

The phenomenon of insecticide resistance is particularly striking in Mediterranean countries, where suitable climatic conditions allow year-round crop production and the survival of *B. tabaci* [3, 12]. Highly resistant MED populations have been reported in Crete*,* a major vegetable crop production area in Greece, especially towards the neonicotinoid insecticide imidacloprid [4]. Pymetrozine, pyriproxifen and spiromesifen, have been introduced and used in rotation with neonicotinoids, alongside an extensive use of natural enemies in whitefly control programs in the last 5 years in Crete. However, a recent resistance monitoring study from various locations in Southern Crete in 2012, showed that neonicotinoid resistance is still present in Crete [13].

The *B. tabaci* cytochome P450 CYP6CM1 was found to be over-expressed in Cretan populations; however, in some cases resistance ratios were not strongly correlated with CYP6CM1 expression level, suggesting that additional neonicotinoid resistance mechanisms may operate in Crete [13].

RNA sequencing (RNA-Seq) was the decisive tool to perform *de novo* transcriptome sequencing and has already been applied in transcriptome analysis of insects, such as *Aedes aegypti*, *Anopheles funestus*, *Nilaparvata lugens*, *Locusta migratoria*, *B. tabaci*, *Trialeurodes vaporariorum* [14–19] and mites [20], providing not only a better understanding of their biological functions at the molecular level but also genomic recourses on detoxification and target genes putatively involved in insecticide resistance [19, 21]. Some recent studies have utilized these techniques to explore differences between resistance phenotypes in insects of medical importance such as *A. aegypti* [22] and *Cimex lectularius* [23]. In *B. tabaci*, there is a number of transcriptome resources generated by Roche 454 pyrosequencing or Illumina deep sequencing which have substantially improved our understanding on topics such as; symbiosis, virus-vector interactions, *Bauveria bassiana* infection, gut role or gene expression in different developmental stages and genetic groups within *B. tabaci* [15, 24–28]. To date only one study dealt with differential gene expression in strains with different resistant phenotypes [29]. Moreover, only a small percentage of the *B. tabaci* transcriptome data available explores the transcriptome of the MED lineage of this complex species [15, 28].

In the present study bioassays and transcriptome data from the MED species were analyzed to further characterize the underlying molecular mechanism responsible for neonicotinoid resistance in *B. tabaci* from Crete. Twelve libraries from three resistant and one susceptible *B. tabaci* strain including biological replicates were sequenced with the Illumina platform, and generated about 73 Gbp of sequence data. This high throughput sequencing technique has allowed for global expression profiling of neonicotinoid resistant and susceptible populations. A reference transcriptome was built by *de novo* assembly and functionally annotated. It was then used to map the reads from insecticide-susceptible and resistant strains, study the levels of gene expression and gain genetic information for resistance mechanisms.

## Results and Discussion

### Resistant phenotypes towards neonicotinoids

Based on recent resistance monitoring in Cretan *B. tabaci* MED, we chose the whitefly populations used in the present study from those displaying the highest resistance phenotypes and heaviest neonicotinoid treatment history. Three field derived strains originally collected from eggplants maintained in the laboratory on cotton plants under Imidacloprid (GR0-IS, GR9-IS) or Acetamiprid Selection (GR4-AS) for few (six) generations were used in this study, along with their Parental ‘relaxed’ strains (GR0-IP, GR9-IP and GR4-AP), namely the respective original strains maintained in the absence of any insecticide contact. All comparisons were performed with a local susceptible reference strain (S-GR6), collected in Crete in 2006, in order to access differences at the transcription level linked with differences at the resistance phenotypes.

Mortality data and Resistance Ratios (RR) to the three neonicotinoid insecticides imidacloprid, acetamiprid and thiachloprid were determined by classical leaf dip bioassays on adult females (Table 1). Compared to the reference susceptible strain S-GR6, RR ranged from 9 to 56-fold for imidacloprid, from 4 to 20-fold for acetamiprid and > 1000-fold for thiacloprid. Resistance ratios in the presence of piperonyl butoxide (PBO), an inhibitor of monooxygenases, were also investigated. Imidacloprid toxicity was increased in all strains (lethal concentration - LC_50_ was reduced from 423 to 185 mg/L for GR4-AS, from 301 to 207 mg/L for GR9-IS and from 177 to 52 mg/L for GR0-IS) after pre-exposure to PBO (Table 1), indicating that cytochrome P450(s) are involved in the reduced susceptibility against imidacloprid. The absence of complete suppression of resistance after pre-exposure to PBO treatment could indicate either the involvement of another resistance mechanism or the limited capacity of the use of PBO to detect P450 based insecticide resistance [30].

**Table 1:** The neonicotinoid response of the susceptible S-GR6 and the resistant *B. tabaci* laboratory strains. Toxicological data to the neonicotinoids imidacloprid, acetamiprid and thiacloprid and toxicity of imidacloprid after pre-exposure to synergist piperonyl butoxide (PBO)

Strain	*n*	LC50 (95 % CI)	RR (95 % CI)	SR (95 % CI)
IMIDACLOPRID				
S-GR6	580	7.5 (5.4–10)	-	
S-GR6 + PBO	543	6.0 (4.3–7.1)	-	-
GR4_AP	586	69 (46–98)	9 (6–13)	
GR4_AS	479	423 (258–657)	56 (36–87)	
GR4_AS + PBO	433	185 (67–302)	30 (17–38)	1.9 (1.7–2.3)
GR9_IP	547	176 (82–268)	23 (15–35)	
GR9_IS	479	301 (186–443)	40 (28–58)	
GR9_IS + PBO	435	207 (164–332)	34 (25–50)	1.2 (1.1–1.4)
GR0_IP	699	82 (60–106)	11 (7–16)	
GR0_IS	451	177 (55–336)	24 (14–39)	
GR0_IS + PBO	425	52 (10–148)	9 (3–16)	2.7 (2.3–3.1
ACETAMIPRID				
S-GR6	503	7.9 (5.1–11)	-	
GR4_AP	442	44 (20–80)	5 (3–9)	
GR4_AS	539	157 (99–242)	20 (15–33)	
GR9_IP	391	56 (28–89)	7 (4.1–11)	
GR9_IS	535	137 (110–163)	17 (11–26)	
GR0_IP	402	33 (25–59)	4 (2.8–6.3)	
GR0_IS	460	71 (42–105)	9 (5.3–15)	
THIACLOPRID				
S-GR6	517	11 (4.2–22)	-	
GR4_AP	422	>30,000	>3,000	
GR4_AS	300	9,740 (835–27,145)	924 (378–2,259)	
GR9_IP	449	>30,000	>3,000	
GR9_IS	320	13,061 (5,253–25,522)	1,239 (720–2,134)	
GR0_IP	422	>30,000	>3,000	
GR0_IS	281	10,477 (3,373–21,100)	994 (457–2,161)	

*RR* resistance ratio at LC50, the Lethal Concentration of 50 %, *CI* confidence intervals resistance characteristics, *SR* synergist ratio: RR/RR after pretreatment with PBO

Compared to their relaxed parental strains, the respective selected strains exhibited higher resistance levels towards acetamiprid and imidacloprid but not towards thiacloprid, indicating that the mechanism underlying resitance to thiacloprid is not selected by imidacloprid or acetamiprid treatments.

The higher resistance levels against neonicotinoids were exhibited by strains GR4-AS and GR9-IS, each selected with a different neonicotinoid (acetamiprid and imidacloprid respectively). These two strains were therefore chosen for styding their transcriptomic profile in the RNA-seq experiment in comparison to the susceptible S-GR6, which was used as a reference strain. In order to better understand the part of *B. tabaci* transcriptom changes linked to the resistance, we compared also strains with the same genetic background which differ only in their resistant level. The pair GR4-AP and GR4-AS displayed the higher differences (6× with imidacloprid and 3.6× with acetamiprid) among the three selected-parental pairs of strains (Table 1). Therefore the parental strain GR4-AP was also included in the RNA-seq experiment.

### Pre-processing treatment reads assembly and functional annotation

Illumina Hiseq 2000 sequencing of 12 cDNA libraries (herein called samples) from four *B. tabaci* strains (3 biological replicates per strain) yielded more than 396 million paired-end reads. The pre-processing technique (low quality nucleotide reads trimming, removal of adaptors, low complexity sequences, poly A/T tail) resulted to 369.04 million reads (72.75Gbp).

The short read sequences were deposited into the Sequence Read Archive (SRA), (BioProject ID: PRJNA293094). The full list of transcript sequences is available upon request from the corresponding author.

The number of reads was evenly distributed among the 12 libraries, with an average of 30.75 +/− 2.4 million reads. The summary of reads per library used in the subsequent analysis is shown in Additional file 1: Table S1.

The intra- and inter-sample fraction for the contaminant ribosomal RNA as well as the mitochondrial cDNA was also similar among them (5.3 % +/− 0.9, 1 % +/− 0.4 and 3.9 % +/− 0.70 for total rRNA, hemiptera rRNA only and mitochondrial RNA, respectively) indicating moderate variation between libraries.

*De novo* assembly performed using Trinity produced 170,377 contigs (mean length: 1136 bp, N50: 2681 bp, Additional file 2: Table S2) corresponding to 113,450 unigenes (a set of contigs which are believed to belong together).

To evaluate the accuracy of the assembled sequences (transcripts), all the usable sequencing reads were aligned onto the transcripts using Bowtie2. A high percentage of reads (97.14 %) were successfully back-mapped on this assembly and at least 71.6 % of the mapped reads were proper pairs.

Similarity between the Trinity assembly and *nr* NCBI protein database was examined using Blastx and disclosed 31,505 contigs with coverage higher than 80 %. In addition, similarity to a dataset of 129 complete mRNA of *B. tabaci* downloaded from NCBI, using blastn, disclosed 134 contigs and 106 *B. tabaci* mRNA sequences that met the criteria initially set (>95 % identity, coverage of the contig or subject 90 % [31]). The average identity was 98.37 %, similar to the level of sequencing accuracy already reported for Illumina technology [31].

The blastx search (*e*-value cut-off <10^−6^) returned 37,393 contigs with at least one blast hit. Additionally, 1,988 contigs were found with blastn performed on the remaining (with no blastx hit) contigs (*e*-value cut-off <10^−10^) (Table 2). InterPro Scan returned 67,687 contigs with a protein domain match.

**Table 2:** Annotation summary

	Contigs	% contigs	Unigenes	% unigenes
	170,377		113,450	
BLAST	39,381	23.11	16,126	14.21
InterPro	67,687	39.73	38,011	33.50
With IPR number	32,945	19.34	12,866	11.34
With > =1GO	25,270	14.83	10,104	8.91
Blast2Go annotated	25,392	14.90	10,685	9.42
EC	6,994	4.02	3,230	2.85
KEGG 137 pathways	6,731	3.95	1,513^a^	1.33

^a^Unique unigenes

To identify the possible functions, Gene Ontology (GO) assignments were used to classify the sequences. A total of 25,392 contigs with at least one GO term (Table 2) were found. The sequences were categorized to ten molecular function (MF), thirteen biological process (BP) and seven cellular unigenes (CC) categories in GO level 2 (general function categories) (Additional file 3: Figure S1). The GO classification results are in line with previous sequenced transcriptomes of *B. tabaci* [15, 32], *T. vaporariorum* [19] and *B. oleae* [33] where binding, cell, catalytic activity and metabolic process were the four largest groups.

The potential enzymes were characterized based on the chemical reaction they catalyze using the prediction of Enzyme Commission (EC) numbers for each sequence. Among the 37,393 annotated contigs, 6,994 contigs were classified to 795 different EC numbers. Enzyme classification revealed that transferases are the largest group of *B. tabaci* MED enzymes (34 %, 269 enzymes) followed by oxydoreductases (22 %, 178 enzymes), hydrolases (22 %, 176 enzymes), lyases (10 %, 76 enzymes) ligases (8 %, 65 enzymes) and isomerases (4 %, 31 enzymes).

To identify the biological pathways that are active in the *B. tabaci* MED, we mapped all contigs with EC number (3,230 unigenes) to the reference canonical pathways in Kyoto Encyclopedia of Gene and Genome (KEGG). In total, we assigned 6,731 (out of 6,994) contigs to 137 KEGG pathways. The pathways mostly represented by the assembled sequences were purine metabolism (52 enzymes, 624 contigs), arginine and proline metabolism (29 enzymes, 95 contigs) and pyrimidine metabolism (28 enzymes, 174 contigs). Contigs for pathways that could be involved in detoxification like metabolism of xenobiotics by cytochrome P450 (7 enzymes, 59 contigs), drug metabolism - cytochrome P450 (6 enzymes, 51 contigs), drug metabolism - other enzymes (15 enzymes, 77 contigs), oxidative phosphorylation (7 enzymes, 113 contigs), glutathione metabolism (15 enzymes, 88 contigs) and nicotinate and nicotinamide metabolism (11 enzymes, 78 contigs) were also represented (Additional file 4: Table S3).

Sequences encoding the targets of the major classes of synthetic insecticides as well as enzymes potentially involved in the xenobiotic detoxification were extracted and compared with sequences from the NCBI protein database. Genes putatively involved in the insecticide metabolic resistance are summarized in Table 3, including conventional detoxification enzymes such as cytochrome P450 monooxygenases (P450s, 146 unigenes), carboxylesterases (CCEs, 23 unigenes) glutathione S-transferases (GSTs, 18 unigenes), UDP-glycosyltranferases (UGTs, 35 unigenes) and ABC transporters (35 unigenes). Putative insecticide targets including nicotinic acetylcholine receptor (nAChR, 10 unigenes), gamma-aminobutyric acid receptor (GABA, 7 unigenes) and acetylcholinesterase (AChE, 3 unigenes) are also shown in Table 3.

**Table 3:** Summary of contigs putatively involved in insecticide resistance

Family	Contigs	Unigenes	Hits	Average contig length (bp)	Average aminoacid length (bp)
Detoxification					
ABC transporter	82	35	36	3,557.9	689.3
CCE	59	23	11	1,816.8	419.0
CPR	2	1	1	4,018.5	694.0
Esterase	152	51	58	3,741.2	537.6
Glutathione peroxidase	1	1	1	2,100.0	204.0
GST	52	18	19	1,512.9	232.7
Hydrolase	136	68	71	3,242.4	490.6
P450^a^	367	146	91	1,764.6	364.1
Peroxidase	13	8	8	4,125.4	701.0
UGT	69	35	22	1,791.5	377.7
Target					
ACCase	1	1	1	5,633.0	1,751.0
AChE	5	3	3	2,603.8	500.4
Chitin synthase	1	1	1	5,600.0	1,571.0
Chloride channels	58	10	15	3,037.4	527.5
GABA	17	7	7	1,971.9	431.1
GluCl	12	2	3	1,676.2	383.2
nAChR^b^	28	10	10	1,872.9	414.1
Ryanodine receptor	1	2	1	15,901.0	3,833.0
Sodium channel	41	12	11	2,303.8	624.7
Other					
Cuticular protein	32	17	16	1,470.6	272.6
Cytochrome b5 reductase	3	3	3	2,377.7	405.3
Catalase	7	4	5	2,672.1	452.2
Superoxide dismutase	11	5	6	1,702.0	208.9
NADH dehydrogenase	38	34	33	1,358.3	136.1
NADH oxidoreductase	4	4	4	1,680.8	298.3

First blast hit, E-value cut-off <10E-10

^a^First tblastn hit against insect P450 proteins

^b^First tblastn hit against insect nAChR proteins

### Investigating target-nicotinic acetylcholine receptors- mediated resistance

In other insect species, mutations as well as alterations in the expression levels of nAChRs, the target of neonicotinoids, have been associated with neonicotinoid resistance [9]. Focused on these genes, we performed a phylogenetic study and investigated sequence polymorphism and expression levels of the nAChRs.

Nicotinic acetylcholine receptors (nAChR) are ligand gated ion channels (LGIC) and serve an important role in nerve signaling, at the post-synaptic membrane, by mediating the fast actions of acetylcholine (ACh) at cholinergic synapses. Neonicotinoid insecticides and spinosad have high and selective affinity for many arthropod nAChRs [34].

**Fig. 1:**
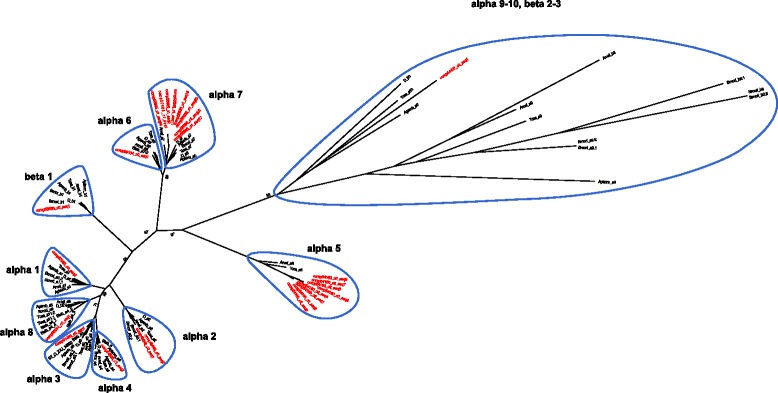
Phylogenetic analysis of *B. tabaci* nAChR subunits. Maximum likelihood phylogenetic tree of *B. tabaci Med* (BtMed, indicated in red) and nAChR subunit protein sequences from 7 species: *D. Melanogaster* (Dmel), *A. mellifera* (Amel), *T. castaneum* (Tcas), *A. gambiae* (Agamb), *B. mori* (Bmori), *A. pisum* (Apisum), *B. tabaci* (Btab). Sequences were aligned using mafft and a bootstrapped midpoint-rooted tree was constructed (1000 bootstraps). Bootstrap percentage is indicated for crucial branches. For accession numbers of insects nAChR subunit protein sequences see Additional file 5: Figure S2

The phylogenetic analysis including 25 contigs and 97 nAChR protein sequences from other insect species revealed 10 nAChR orthologs in *B. tabaci* MED encoding putative receptor subunits (Fig. 1, and for alignment Additional file 5: Figure S2). A comparative number of subunits (10 and 12 nAChR subunits) was found in *A. pisum, An. gambiae*, *Ap. mellifera*, *B. mori*, *D. melanogaster* and *T. castaneum.* Nine nAChR subunits, α1, α2, α3, α4, α5, α6, α7, α8, and β1 were grouped into highly conserved core groups and were named accordingly [35]. Interestingly, as in other insects, *B. tabaci* contained a subunit which showed particularly low sequence identity with other nACh receptors and was clustered into a divergent group including subunits α9 and β2 of *A. mellifera*, β3 of *D. melanogaster*, α9 and α10 of *T. castaneum*. The bidirectional best hits of this subunit against all insects and acari nAChR are for *Lοcusta migratoria* α9 (46 % similarity) and *T. castaneum* α10 (44 %). However, in this subunit one of the two vicinal cysteines of the motif YxCC at Loop C, defining α subunits, was replaced by a histidine and is therefore named β2 (Additional file 6: Figure S3).

Sequence comparison results show that most of the *B. tabaci* nAChR subunits contained a GEK motif preceding TM2 (Additional file 6: Figure S3), a highly conserved motif in most insect nAChR subunits where the glutamate residue is considered to be associated with cation selectivity. However, the GEK motif was replaced by REK in α2 and by ΜΕR in β2. Such results have been also observed in *B. mori* where the motif was replaced by STR in α9 and β3, and by TIR in β2 [36]. Whether *B. tabaci* MED α2 and β2 possess the cation selectivity as other GEK-motif-containing AChR subunits remains to be determined.

It is worth noting that two subunits were represented by variant isoforms: α4 and α5 with 2 and 3 variants respectively. Alternative splicing occurs commonly and has been observed in the α3, α4, α6, α7 subunits in many insects including *A. melifera* [37], *A. pisum* [38], *B. mori* [36], *T. castaneum* [39]. It effectively diversifies receptor function and generates species-specific isoforms by introducing variations in the Cys-loop that are involved in receptor assembly, in loops E and B. These variations contribute to ligand binding in the second transmembrane domain, which forms the ion channel between TM2 and TM3, that are important in coupling agonist binding to ion channel gating [40, 41]. Together with alternative splicing, post transcriptional RNA A to I editing (especially in α 6 subunit) and other alterations like truncated transcripts (α7 and α4) contribute in largely increase the diversification of nACh receptors between insect species [35, 39, 42–44] and counterpart the low number of insect subunits compared to vertebrate nAChR. Whether the different isoforms observed in nAChR subunits α4 and α5 of *B. tabaci* consist of alternative transcripts or not, clearly warrant further investigation by combining transcriptomic and genomic data.

**Fig. 2:**
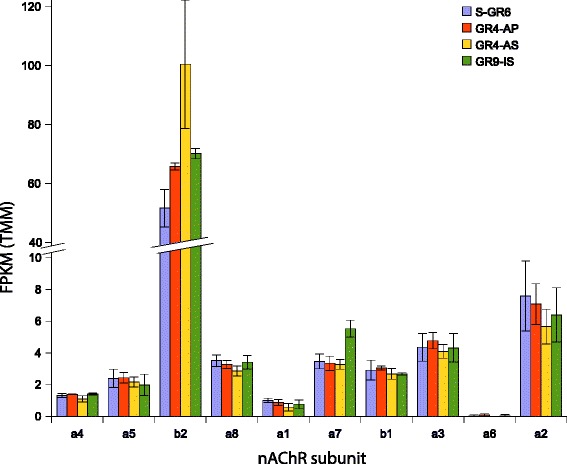
Graph bar diagram of FPKM normalized (TMM) expression level of *B. tabaci* nAChR subunits

We found substantial variation in expression levels between the different subunits of nACh receptors. In all strains, subunit β2 was the most highly expressed (average FPKM across libraries = 52 to 100) followed by the α2 (FPKM = 6 to 8) and the similarly expressed α3, α7 and α8 (FPKM = 3 to 6). The lower expressed subunit was α 6 with only ten mapped reads (Fig. 2). We also examined if differential gene expression occurred between strains. DE was found for β2 between S-GR6 and GR4-AP (logFC = 0.94, FDR = 9.2 10^−5^) or GR4-AS (logFC = 0.60, FDR = 0.032) and for subunit α7 between S-GR6 and GR9-IS (logFC = 0.65, FDR = 0.011). Whether this differential gene expression between resistant and susceptible strains for subunits β2 and α7 contributes to the observed resistance phenotypes needs further examination. However, it is interesting to point to recent studies that established a link between nAChR expression and response to neonicotinoids. Taillebois et al. [45] showed that pre-exposure to different neonicotinoids significantly affected the expression levels of some subunits which were depended on the nature of the insecticide. The importance of differential expression of subunits in the regulation of neonicotinoid sensitivity is also suggested by the findings of Yu et al. [46], who observed a significant decrease of expression of β1 and β2 after exposure to imidacloprid. Moreover, Markussen and Kristensen [47] found that neonicotinoid resistance phenotype in Danish *Musca domestica* was correlated with reduced expression of the nAChR subunit α2.

We investigated the occurrence of SNPs in unigenes encoding nAChRs and possible differences between resistant and susceptible *B. tabaci* strains. A total of 347 SNPs were identified and were equally distributed among strains (283 in S-GR6, 324 in GR9-IS, 297 in GR4-AS and 310 in GR4-AP). Most of them (52 %) were found in the coding regions, 19 % in the 5′UTR and 29 % in the 3′UTR.

The number of SNPs differed between the subunits of nAChR with the subunits α8 and β1 showing higher variation (111 and 68 SNPs, respectively) and subunits α5 and α7 lower variation (only 1 and 2 SNPs, respectively). In subunit α6, no variable site was detected; however, the shorter transcript was recovered for this subunit.

Comparative polymorphism analysis identified several alleles differentially represented between insecticide resistance strains and the reference susceptible strain (>50 % allele frequency difference, referred to as ‘differential SNPs’). Twenty-seven out of the 347 SNPs were differential SNPs (10 in the coding region and 17 in the 5′UTR or in the 3′UTR). Seventeen polymorphisms concerned SNPs with high frequency differences between the S-GR6 and any of the resistant strains, which were equally affected (15, 15 and 16 differential SNPs for GR9-IS, GR4-AP and GR4-AS, respectively). Only five differential SNPs between S and R strains were in the coding region and all of them concerned synonymous substitutions.

Among all transcripts, five out of the 347 SNPs annotated in the nAChR sequence assembly were found in subunits β2 (3), α3 and α4, resulted in an amino acid change (Additional file 6: Figure S3). In subunit β2, one non-synonymous SNP (nsSNP) was found at the N terminal signal peptide and involved an alteration of F and S residue at position 12 (aa numbering as in alignment of Additional file 6: Figure S3) and two others, D/E and N/T at positions 400 and 426, were found between transmembrane domain TM3 and TM4. In subunit α3, a nsSNP (P/A) was found at position 448 between TM3 and TM4. Finally, an F/Y change has been detected in subunit α4 located at 159 residues between LpA and LpE. None of the mutations leading to substitutions known to confer target resistance to neonicotinoids in other insect pests were detected (coverage 65 to 309 for the specific sites). Despite the apparent scarcity of target site resistance to neonicotinoids compared to increased detoxification, two target mutations conferring resistance to neonicotinoids have been documented in other agricultural pests [9, 48]. An amino acid substitution in a highly conserved site of the loop B of two nAChR subunits (α1 and α3), that causes a tyrosine to serine substitution (Y151S), was detected in the brown planthopper *Nilaparvata lugens* conferring imidacloprid resistance [9]. The apparent scarcity of this mutation, in any other insect species including neonicotinoid resistant *B. tabaci,* could be explained (as discussed by Liu et al. [9]) by the hypothesis that insects may need two mutated subunits to express resistance. Another mutation in a highly conserved site within loop D of β1, that causes an arginine to threonine substitution (R81T), was found in highly resistant clones of the aphid *Myzus persicae* [48]*.* In addition, the substitutions found in our study were not located in any of the hot spot regions of the nAChR subunits i.e. loops D, E and F of subunit β1, or loops A, B and C of α subunits. These regions form the binding site for acetylcholine and for certain agonists including neonicotinoids [49], where a number of key residues for toxin and neonicotinoid binding and selective toxicity in insect nAChR have been identified by functional expression of hybrid or chimeric receptors and by docking simulations (reviewed in [35, 50]).

### Investigation of non - target site resistance mechanisms based on differential expression

We compared gene expressions of the susceptible and the three neonicotinoid resistant strains and performed differential expression analysis (DE). Data of three biological replicates of each condition were used in this analysis (in total twelve samples). The biological coefficients of variation (BCV) observed in this study ranged from 0.11 to 0.19 (BCV: GR4-AS = 0.19, GR4-AP = 0.11, GR9-IS = 0.15, S-GR6 = 0.17, mean 0.16 SE = 0.018) and are close to those observed in genetically identical model organisms, indicating small variability between replicates [51]. The low intra biological replicate variation and the clear separation between susceptible and the three resistant strains provided a solid base to all subsequent analyses (Additional file 7: Figure S4).

The DE analysis revealed in total 3,745 differentially expressed unigenes (|logFC| >1, FDR < 0.05) in the three R strains compared to the S strain based on their TMM-FPKM (Additional file 8: Table S4). According to the expression levels of the 3,745 DE unigenes we examined the overall differential expression and detected three sub-clusters that exhibited marked differences in normalized FPKM. This clustering grouped the neonicotinoid resistant samples together. Assigning known transcripts to molecular functions revealed differences in the GO enrichment in the three clusters, showing that substantial differences in the transcriptional profile between resistant and susceptible strains exist (Additional file 9: Figure S5).

**Fig. 3:**
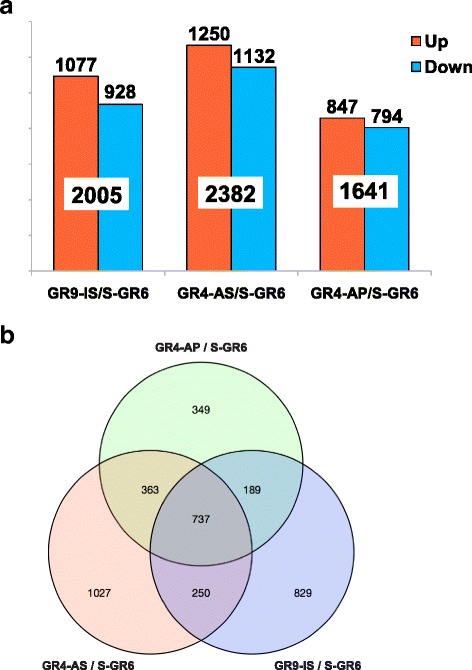
Graph bars and venn diagram of differentially expressed unigenes of the three resistant strains. **a)** Distribution of differentially expressed unigenes of the resistant strains compared to the susceptible strain (log2FC >1, FDR < 0.05). In each strain up and down regulated unigenes are indicated. **b)** Venn diagram of differentially expressed unigenes in the three resistant strains. For each Venn diagram section, the numbers of transcripts differentially expressed in any insecticide resistant strain as compared to the susceptible strain are indicated

The frequency of the up- and down-regulated unigenes was not significantly different within each strain. Differential transcription was homogenously distributed between up- and down-regulated unigenes in all strains with an almost 50 % frequency: 1,077, 1,250 and 847 unigenes were up regulated and 928, 1,132 and 794 unigenes down regulated in GR9-IS, GR4-AS and the GR4-AP, respectively, compared to the susceptible S-GR6 strain (|logFC| >1, FDR < 0.05) (Fig. 3a).

The total number of DE unigenes against S-GR6 was unbalanced (Fig. 3) suggesting a quantitative difference in transcription response between strains, while the lower number of DE unigenes was found in GR4-AP. A total of 1,539 unigenes were found differentially expressed in any strain, including 332 and 405 unigenes over and under transcribed in all strains, respectively. The two selected strains (GR4-AS and GR9-IS) showed the higher number of DE unigenes with 584 and 528 transcripts specifically over-transcribed as compared to the susceptible strain (Fig. 3b). In contrast, fewer transcripts were differentially expressed in the parental strain (GR4-AP), maintained with no selection, with only 212 specifically over transcribed genes. Not surprisingly, the comparison between GR4-AS versus GR4-AP revealed the lower number of regulated genes, 159 up- and 167 down-regulated unigenes (Fig. 3b).

**Fig. 4:**
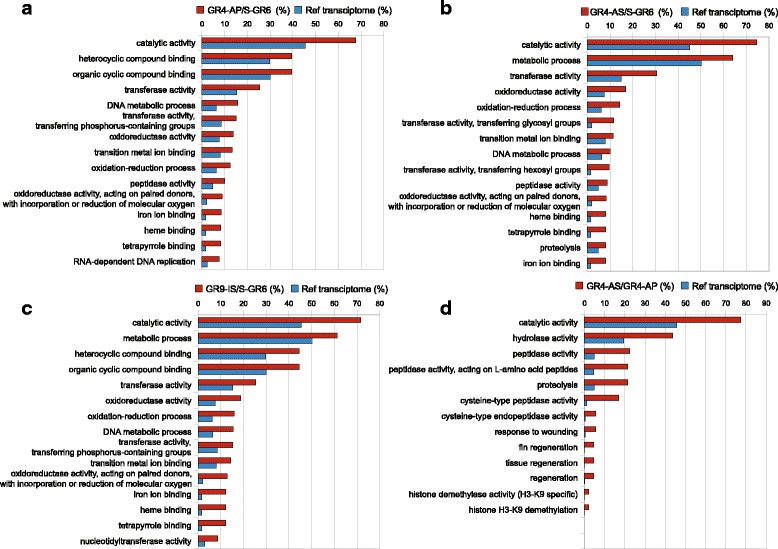
GO bar diagrams of over-represented and up-regulated genes. Each diagram shows the first 15 GO terms over-represented and up-regulated genes in the three resistant strains vs S-GR6 (**a**, **b** and **c**) and the first 13 GO terms in GR4-AS vs GR4-AP (**d**). Fisher’s exact test (adjust *p*-value <0.05) on the GO terms

For a better understanding of the molecular functions and biological processes underlying the nature of resistance to insecticides, Gene Ontology term enrichment analysis was used to detect differentially expressed unigenes over or under represented specifically in each strain. Here, the term enrichment results for the comparison between the DE unigenes of each strain against the reference transcriptome. For the up-regulated unigenes, the GO terms were generally negatively enriched in the resistant strains including terms such as response to stimulus (GO:0050896), signalling (GO:0023052) and cellular process (GO:0009987) in all three resistant strains and terms such as binding (GO:0005488) only in GR4_AS. Among the higher significant positively enriched GO terms corresponding to up-regulated unigenes figured those related to catalytic activity such as transferase (GO:0016740) and oxidoreductase activity (GO:0016491) in all three resistant strains together with terms related to heterocyclic compound binding (GO:1901363) and organic cyclic compound binding (GO:0097159) in GR4_AP and GR9_IS (Additional file 10: Table S5, Fig. 4).

In genes with up-regulation in the GR4_AS compared to its parental GR4_AP hydrolase (GO:0016787) and peptidase activity (GO:0008233) are among the terms related with catalytic activity (GO:0003824), for which the frequency increased significantly (Fig. 4). Interestingly, among the down-regulated unigenes the term “response to stress” (GO:0006950) figures among the over represented terms suggesting that genes related to stress are not only down-regulated in GR4-AS compared to the parental but also represent a higher percentage of total GO terms down regulated compared to the reference transcriptome (Additional file 10: Table S5).

In addition to the differential gene expression of nAChR subunits between resistant and susceptible strains that was examined in the “target” paragraph, the expression profiles of other genes known to be involved in insecticide resistance were closely examined (logFC >1, FDR < 0.05) (Additional file 8: Table S4). This DE analyses includes genes related to insecticide detoxification or altered insecticide transport and sequestration.

Among the 550 differentially over-transcribed unigenes in any R strain with a Blast hit, several genes related to altered insecticide transport and sequestration were found including five cuticular proteins (one unigene with logFC 2.48 and 4.27 in AS and AP, respectively; GO:0042302) and five fatty acid synthases (Additional file 8: Table S4). Typically, the metabolic detoxification system in insects consists of enzymes, acting on a broad range of substrates directly to reduce their toxicity, represented by cytochrome P450 monooxygenases (P450s), and carboxylesterases (CCEs) or to facilitate the excretion of hydrophobic toxic compounds by improving their hydrophilicity such as glutathione-S-transferases (GSTs) and UDP-glucuronosyltransferases (UGTs). The strong response of the GR9-IS, GR4-AS and GR4-AP to insecticide selection through transcription level modifications was confirmed. Among the 550 differentially over expressed unigenes in any R strain with a Blast hit, several detoxification enzymes were represented including unigenes with orthologs in P450s (35 unigenes), GSTs (2), CCEs (8) and UDP-glucuronosyltransferases (7) with some of them, namely P450s, displaying a logFC > 5 (Additional file 11: Table S6).

The between strains comparison displayed that, compared to the susceptible strain S-GR6, the number of the up-regulated DE detoxification unigenes (36, 32 and 18 in GR4-AS, GR4-IS and GR4-AP, respectively; Additional file 11: Table S6) differed significantly (Fisher exact test) between the GR4-AP and either GR4-AS or GR9-IS, but not between GR9-IS and GR4-AS. Moreover, this number is in accordance with the resistance ratios observed in the three neonicotinoid resistant strains (Table 1) suggesting a quantitatively differentiated transcriptional profile related to insecticide detoxification.

GR4-AP shared all of its up regulated detox unigenes with at least one of the selected strains. The 13 unigenes shared by all resistant strains (including the parental relaxed strain) should represent genes selected by neonicotinoid or other insecticides applied in the field and maintained their expression levels in GR4-AP strain even after 6 generations without insecticide exposure. The four significantly up regulated unigenes shared only by the GR9-IS and GR4-AS, which may have been commonly selected by either neonicotinoids, include three P450s and one CCE with moderate logFC ranging from 1 to 2 (Additional file 11: Table S6).

Investigation of detoxification genes specifically over-expressed in each resistant strain disclosed that two CCEs, one UGT, and 11 P450s (in total 14) were specifically up regulated in GR9-IS strain, with one CCE and one P450 having the higher logFC (2.7 and 3.7 respectively). Similarly, 17 unigenes were specifically up regulated in GR4-AS including 10 P450, 4 UGTs, 2 GSTs and 1 CCE. These specifically GR9-IS or GR4-AS up regulated unigenes reflect a genetic background related specificity, a selection agent (imidacloprid or acetamiprid) specificity, or a combination of both. Our experimental design does not allow distinguishing between the two cases. However, taking into account that these strains should belong to the same genetic cluster (south Crete) identified by microsatellites [52], it is possible that the different (specific) up-regulated unigenes in each GR9-IS or GR4-AS strain reflect insecticide specificity. One P450 unigene (comp43065_c0) was up-regulated in the GR4-AS compared to both S-GR6 and its parental strain GR4-AP (logFC > 2.5). This unigene might represent a gene with a positive transcription reaction to the selection with acetamiprid and should be studied further.

GSTs metabolism as a mechanism of resistance to organochlorines, carbamates, organophosphates and pyrethroids has been reported in many arthropod species [53–56]. Similarly enhanced production of carboxylesterases through gene amplification and/or up-regulation has been demonstrated in several organophosphate, carbamate or pyrethroids resistant insects [57–59]. However, GSTs or COE have never been directly associated with neonicotinoid resistance and therefore the over-expression of the GSTs and COEs in the GR9_IS, GR4_AP and GR4_AS may have been selected as a result of contact with other xenobiotics in their environment.

Among detoxification enzyme systems, P450s are the most common genes found up-regulated in a wide number of resistant insects with a subset of these P450s confirmed to be able to metabolize pesticides by functional in vitro studies in mosquitoes species reviewed by [60, 61], *M. domestica* [62], *D. melanogaster* [63], *N. lugens* [64], *H. armigera* [65, 66], *T. urticae* [67, 68] and *B. tabaci* [10, 69, 70]. In addition, monooxygenase-mediated neonicotinoid resistance has been reported in several insect species including *B. tabaci* [11, 21, 29, 71, 72], *M. domestica* [73], *M. persicae* [74] and *D. melanogaster* [75]. P450s expression profile is examined in more details in the following paragraphs.

### Detailed study of transcripts encoding putative P450s

#### Phylogenetic study of transcripts encoding putative P450s

P450s are one of the largest super-families, playing a dominant role in plant-insect interactions and insecticide/xenobiotic metabolism. They are divided in 4 major clades, named CYP2, CYP3, CYP4 and mitochondrial [30]. A variable number of P450s has been identified in insect genomes such as *D. melanogaster* (88), *A. gambiae* (105), *A. aegypti* (160), *T. castaneum* (134), *A. pisum* (64) (reviewed in [30]) and in recent transcriptome studies of *B. dorsalis* (90), *B. oleae* (55) and *T. vaporariorum* (57) [19, 31, 33].

A total of 367 contigs (146 unigenes) were identified as P450 encoding genes in the *B. tabaci* transcriptome (Additional file 12: Table S7). To assign *B. tabaci* P450s to respective P450 clusters and families, a phylogenetic analysis was performed and, in the case of misaligning protein sequences, the closest blastp hits in the NCBI *nr* database were identified. In the phylogenetic analyses, only the longest 171 non-redundant contigs (115 unigenes) were included together with other known insect P450s (345 protein sequences from 14 species including *B. tabaci*).

In this dataset, representatives of all 4 major insect P450 clades were found. The majority of *B. tabaci* P450s (76 out of 171) belonged to the CYP3 clade, 73 to the CYP4, 18 to the CYP2, and 4 to the mitochondrial clade (Additional file 13: Figure S6). Most of *B. tabaci* P450s included in CYP3 and CYP4 clades cluster separately from P450s from other insect species of the phylogenetic analysis. In CYP4, the separate clusters formed by *B. tabaci* P450s are supported by high bootstrap values and most of them form sister groups to other Hemiptera P450s (ex *A. pisum*). However, this separation is not as clear in CYP3 *B. tabaci* P450s.

Finally, we performed a Bidirectional Best Hit (BBH) with 1:1 relationship between the 171 P450s contigs of the current study and the 345 P450 proteins from different species that were also included in the phylogenetic analysis. The BBH returned 35 P450 orthologs. For the remaining P450s, a corresponding name was assigned according to the first Blast hit. (Additional file 12: Table S7).

#### Expression levels of transcripts encoding putative P450s

The phylogenetic analyses showed that most of the 35 up-regulated P450 unigenes in any *B. tabaci* strain belonged to the two larger P450 clades CYP4 and CYP3, with 18 and 15 unigenes, respectively. Three unigenes belonged to CYP2 and only one in the mitochondrial clade. More specifically, most of them corresponded to genes from CYP6, CYP4 and CYP417 families, but also from CYP303, CYP301, CYP304 and CYP439 families (Additional file 12: Table S7). We subsequently focused on the higher over-expressed P450 unigenes which displayed logFC > 2 in at least one resistant strain. Eight unigenes that belonged to clades CYP2 (2), CYP3 (3) and CYP4 (3) met this criterion. Six unigenes (comp50040_c0, comp61334_c0 comp62212_c0, comp57969_c113, comp57969_c124 and comp33028_c0) were up regulated in all resistant strains compared to the S-GR6 strain. One unigene, comp43434_c1, was highly up-regulated only in GR9_IS (logFC = 3.7) and another, comp43065_c0, only in the GR4_AS strain (logFC = 5.4) (Additional file 12: Table S7). In all cases, P450 expression was higher in the GR4_AS maintained under acetamiprid selection, compared to its corresponding parental relaxed GR4_AP suggesting the possible contribution in acetamiprid metabolism. However, a significant difference between selected and parental strains has been found only in the comp43065_c0 (Additional file 12: Table S7).

Unigenes comp61334_c0 and comp33028_c0 shared the higher similarity (higher best hit) with a putative P450 CYP303a1-like of *A. pisum* and in the phylogenetic analysis clustered together with CYP303a1 of *A. pisum* and *L. striatella* (100 % bootstrap). They were both significantly up-regulated in the resistant strains compared to the S-GR6 however, comp61334_c0 displayed higher up regulation (logFC = 5.57–6.58) compared to comp33028_c0 (logFC = 1.4–3.2). These two specific P450s belong to clade CYP2 and are presumed 1:1 orthologs with CYP303a1 family members. Interestingly, CYP303A1 is one of few highly conserved P450s found in all insect species sequenced to date, but not in *Daphnia pulex* [30]. To our knowledge members of this family have never been associated with resistance to any insecticide and this is the first report for such a finding. In *D. Melanogaster*, CYP303A1 has a putative external sensory development function and it has been hypothesized that it probably metabolizes a small signal molecule essential in the development and structure of external sensory organs [30, 76]. Whether this has to do with the sensorial capacity and avoidance of treated surface behavior remains unknown.

Components 62212_c0 and 43434_c1 shared the higher similarity with CYP417B1 of *L. stratiella* (orthologous to CYP417). They belong to the CYP4 clade, with which they formed a separate cluster with other P450 unigenes of *B. tabaci* issued from this study. This cluster was a sister group with members of CYP417, CYP425, CYP426 and CYP439 of *L. stratiella*. Comp62212_c0 was highly up-regulated in the three resistant strains (logFC = 4.78–6.07) while comp43434_c1 was up-regulated only in the GR9-IS strain (logFC = 3.73).

A third unigene of clade CYP4, comp43065_c0, shared the higher similarity and was 1:1 ortholog with CYP4C1 of *A. pisum,* with which formed a distinct group separated from other CYP4C1 *A. pisum* sequences. This unigene was highly over-expressed in the GR4-AS strain (logFC = 5.36) and was also significantly increased level of expression after selection with acetamiprid (logFC = 2.45 in GR4-AS compared to GR4-AP). Over-expression of members of CYP4 family has been found associated with a number of insecticide resistant insect strains (reviewed in table 7 of [30], but also [77]) including neonicotinoids. For example, members of CYP4 (CYP4C1 and CYP4v2) family have been found over-expressed in MEAM1 *B. tabaci* resistant to thiamethoxam [29, 32], or induced after treatment with imidacloprid (CYP4G36) in *Aedes aegypti* [78], or in *Diaphorina citri* (CYP4C67, CYP4DA1, CYP4C68, CYP4DB1, and CYP4G70) [79].

Unigene comp50040_c0 had a significant Blast hit with CYP6CM1vQ of *B. tabaci* (100 % similarity) and was differentially unregulated in the three resistant strains with similar, very high expression levels, compared to the susceptible strain (logFC = 5.55–5.84).

CYP6 family P450s are widely known to be involved in metabolic resistance to pyrethroids and organophosphates in a number of insect species [80–83] including neonicotinoids [74, 75, 84]. CYP6CM1 was the first P450 isolated from an agricultural pest to be directly involved in neonicotinoid metabolism by recombinant expression. More specifically, it has been found able to metabolize imidacloprid, clothianidin and thiacloprid but not acetamiprid [10, 69]. It has been also found to metabolize the chemically unrelated insecticides pymetrozine [70] and pyriproxifen [85]. The cross resistance of acetamiprid-selected strain towards imidaclorpid (Table 1) could explain the high expression levels of CYP6CM1 in GR4-AS. Additionally, the comparable expression levels with the relaxed parental strain could mean that this P450 was initially selected in the field (due to selection by other neonicotinoids) and maintained the same expression levels after six generations in absence of any selection.

For components 57969_c113 and 57969_c124 the bidirectional best hits (1:1) were respectively CYP6CX5 and CYP6CX3 of *B. tabaci*. These two unigenes showed comparable over-expression levels in the three resistant strains (logFC = 1.23–2.06 for comp57969_c124 and logFC = 1.61–2.45 for comp57969_c113). Although there is no significant difference, their expression levels slightly increase with acetamiprid selection in GR4_AS compared to GR4_AP. In the phylogenetic analysis, they were grouped together with the third member of clade CYP3 (CYP6CM1) that has also been found highly over-expressed in this study. All three (CYP6CX5, CYP6CX3 and CYP6CM1) displayed the higher similarity with the respective published sequences of *B. tabaci* as well as with CYP6CX1 and CYP6CX4. CYP6CX1 has been associated with *B. tabaci* (biotype B) resistance to fenvalerate, chlopyrifos, and avermectin in the field [86]. CYP6CX1 exhibited significantly higher mRNA expression in a laboratory-selected thiamethoxam resistant *B. tabaci* MEAM1 (B-biotype) strain [71].

All of the eight above mentioned P450s may be potentially associated with insecticide resistance and this could constitute a discrepancy compared to other insects, ie *D. melanogaster* where only a single P450, cyp6g1, is associated with neonicotinoid resistance [75]. However *B. tabaci,* is a heavily treated agricultural pest and different insecticides, of the same or different chemical groups, may select several P450s conferring unpredictable cross resistance. For example, CYP6CM1 the only functionally characterized P450 in *B. tabaci*, confers multiple resistant phenotypes and metabolizes some but not all neonicotinoids, as well as other insecticides from different chemical groups. Our differential expression study provides a good base to select additional candidate genes for further functional characterization and validate their role in resistant phenotypes.

#### P450 expression levels in laboratory and field B. tabaci displaying different resistance phenotype using qPCR

**Fig. 5:**
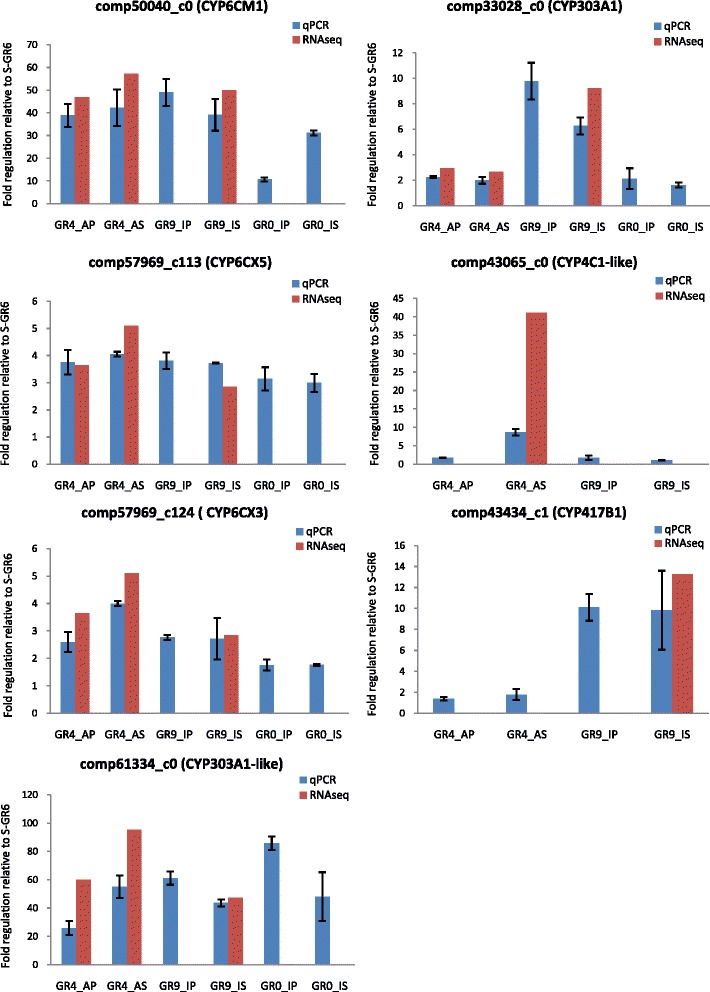
qPCR P450 expression analysis of neonicotinoid resistant strains vs susceptible strain

To further support P450s role as candidate genes we studied the association between resistant phenotype and expression levels. Focusing on P450 enzymes, we were able to set up a quantitative PCR for the seven of the eight higher up-regulated P450s (see previous paragraph) with logFC > 2 (comp50040_c0, comp61334_c0, comp33028_c0, comp57969_c113, comp57969_c124, comp43065_c0 and comp43434_c1). Using this approach, we investigated their expression levels in several selected strains (some of them used in the RNA-seq experiment), as well as in additional whiteflies sampled in the field. The levels of gene expression (folds regulation) in resistant strains compared to the susceptible S_GR6 found in qPCRs were highly correlated with those observed in RNAseq experiment (*R*^2^ = 0.85; *P* < 0.0001) (Fig. 5). This concordance between the transcription profiles obtained by the two techniques offered confidence to use qPCR and interpret the levels of expression of P450s in relation to neonicotinoid selection and resistance phenotype.

We first examined the levels of expression in comparisons between the strains maintained under neoinicotinoid selections and their respective relaxed strains (GR4_AS versus GR4_AP, GR9_IS versus GR9_IP and GR0_IS versus GR0-IP) (Fig. 5). In general, the P450 expression levels were higher in the strain GR4_AS, maintained under acetamiprid treatment, compared to its respective relaxed GR4_AP strain pointing out the involvement of these P450s in acetamiprid resistance. This has not been always the case for the two imidacloprid selected strains. More precisely, in these pair wise comparisons the laboratory acetamiprid treatment differentiated the expression levels for comp57969_c124, comp61334_c0 and comp43065_c0 but left unchanged the levels of CYP6CM1, comp57969_c0, comp 33028 and comp43434 (Fig. 5). On the other hand, the pair wise comparisons disclosed that the imidacloprid treatment left the levels of expression unaffected for most of the unigenes except for CYP6CM1 that was higher in the selected counterpart in one of the two “imidacloprid” strain pairs (GR0_IS versus GR0_IP). This result could be explained by differences in phenotypic profiles toward the two neonicotinoids. Indeed, it is worth noting that although tolerance to both neonicotinoids was constantly higher in the strains maintained under treatment (GR0_AS, GR4_IS and GR9_IS), the confidence intervals (95 % CI) of Resistance Ratio (RR) in the imidacloprid selected strains (_IS) overlap with the CI of RR of their respective relaxed strains (Table 1) to both imidacloprid and acatamiprid. On the other hand, tolerance to acetamiprid and imidacloprid has been significantly higher in the acetamiprid selected strain (absence of CI overlap) compared to the parental one which has been maintained under the same conditions without acetamiprid (and any) treatment.

We subsequently scaled up and examined with qPCR the expression levels of these seven P450s, in five additional whitefly populations directly derived from the field. All populations belonged to MED *B. tabaci* and were collected from eggplants grown in greenhouses in Crete, setting the frequent application of neonicotinoids, pyrethroids, spiromesifen and pymetrozine as the common point in their treatment history. Compared to the laboratory susceptible strain S_GR6, their RR ranged from 6 to 65-fold for imidacloprid and 2 to 26.5-fold for acetamiprid (Additional file 14: Table S8). For three unigenes (com33028_c0, comp43065_c0 and comp43434_c1), relative over-expression of the P450s in the field populations, measured by qPCRs, reached a 2-fold difference compared to S-GR6 while in unigenes comp57969_c113 and comp57969_c124 they increased 5.5-fold. Finally, in comp50040_c0 and comp61334_c0 the higher over-expression was detected with FC starting from 5 and reaching as high as 190-fold compared to the susceptible strain S_GR6 (Additional file 14: Table S8).

**Fig. 6:**
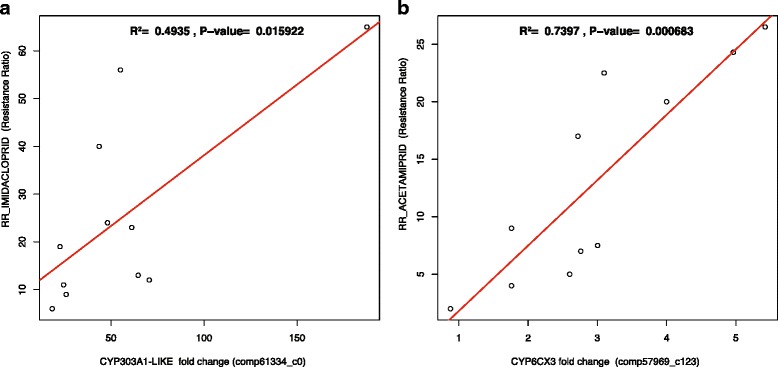
Linear significant regression P450 analysis between resistance ratios and relative gene expression. **a**) Correlation between expression of CYP303A1-LIKE and resistance ratio to imidacloprid, **b**) correlation between expression of CYP6CX3 and resistance ratio to acetamiprid.

We then looked for the presence of correlation between resistant phenotypes and expression levels by plotting RR of all field derived and laboratory maintained strains against qPCR relative fold regulation. A positive correlation was observed between expression levels of comp61334_c0, CYP6CM1 and comp43065_c0 and RR to imidacloprid and between the expression of components 57969_c124 and 57969_c113 and RR to acetamiprid. However, a significant positive correlation has been found only between the expression of comp61334_c0 and RR to imidacloprid, and comp57969_c124 and RR to acetamiprid (*R*^2^ = 0.5, *P* = 0.02; *R*^2^ = 0.7, *P* < 0.001, respectively) (Fig. 6).

In field MED (Q biotype) *B. tabaci* populations from China, levels of resistance to imidacloprid were correlated with expression levels of two P450s: CYP6CM1 and CYP4C64 [72]. In our RNA-seq data, comp47612_c0 with the best Blast hit to the CYP4C64 from *B. tabaci* appeared among the differentially over-expressed P450s in both acetamiprid selected and parental strain (GR4_AP and GR4_AS), with logFC close to 1. For this reason, its expression levels were not examined in more populations by qPCR and we were are not able to assess the presence of any possible correlation with resistance levels in the Greek populations. Components 61334_c0 and 57969_c124 are the number one candidates for further studies, i.e. functional analysis, given their stronger positive correlation with the neonicotinoid resistant phenotype. However, the absence of significant correlation between RR and relative fold regulation cannot exclude the possibility of another P450 being involved in imidacloprid or acetamiprid resistance. Rather, it indicates that this P450 is probably not the major, in terms of frequency, resistance mechanism for the given populations. This is illustrated by the case of CYP6CM1 whose implication in neonicotinoid resistance has been functionally demonstrated; although in the present study, no significant correlation was found. It is worth noting the total absence of a correlation between CYP6CM1 and RR to acetamiprid is in agreement with the fact that acetamiptid is not metabolized by this P450. The unpredictable diverse range of chemistries detoxified by CYP6CM1, which includes some - but not all - neonicotinoids [10, 69] as well as the unrelated chemistries of pymetrozine [70] and pyriproxyfen [85], exemplify the complex relationship between resistance and expression of a single P450. Our study clearly demonstrates that several P450s can simultaneously operate in a given resistant population and that expression profile can change between populations. This suggests that different P450 genes could be involved in different cases of neonicotinoid resistance.

## Conclusion

Our work has generated new toxicological data and genomic resources which will significantly enrich the available dataset and substantially facilitate the molecular studies in MED *B. tabaci*. Twelve libraries from three neonicotinoid resistant *B. tabaci* strains from Greece were sequenced by Illumina platform, and generated about 73 Gbp. A reference transcriptome was built by *de novo* assembly and functionally annotated. The large number of sequences, obtained by the investigation of expression profile of different neonicotinoid resistant strains, provided useful insights into the underlying molecular mechanisms associated with resistance. We further focused on the differential expression analysis and phylogenetic classification of nAChR subunits and cytochrome P450s, the main genes related to neonicotinoid resistance, enabling useful genetic information of resistance mechanisms to be gained. No nAChR polymorphism or differential expression of the different nAChR subunits were observed among the studied strains that could be related to the resistant phenotypes. Differential expression analyses disclosed a number of significantly up regulated detoxification genes (mainly cytochrome P450s), some of which specifically over-expressed in each selected strain, providing a list of putative candidate resistance genes. CYP6CM1 already known as a major mechanism of imidacloprid resistance in *B. tabaci* from Crete was also deferentially expressed in the resistant strains. Using quantitative qPCRs, a larger screening of field MED *B. tabaci* from Crete with known neonicotinoid phenotype was performed to associate expression levels of P450s with resistance levels. Expression levels of five P450s including CYP6CM1 were found associated with neonicotinoid resistance, however, a significant correlation was found only in two of them, CYP303 and CYP6CX3, with imidacloprid and acetamiprid respectively*.* Further functional expression analysis of the genes and the encoded proteins are currently being investigated to confirm their role in resistance.

## Methods

### Sample collection/biological material

Three *B. tabaci* populations (GR0, GR4 and GR9), collected in 2012 in Crete on eggplant greenhouses were maintained in laboratory conditions under short term selections and were chosen based on their LC_50_s (concentrations that cause 50 % mortality) to neonicotinoids and their insecticide application history. Insecticide selection experiments were carried out in parallel for the three strains during six generations with the neonicotinoids acetamiprid (GR4_AS) and imidacloprid (GR9-IS and GR0-IS). During the experiment, the initial parental strains (GR4-AP, GR9-IP and GR0-IP) were also maintained without insecticide selection. Under these relaxed conditions, it is expected that the Resistance Ratio (RR) in the parental strains would be lower than the respective selected ones, given that a fitness cost is often associated to the insecticide resistance.

Five additional *B. tabaci* populations collected in Crete in 2013 from eggplants grown in greenhouses, were also used in our study without any prior rearing in the laboratory (BT1, BT2, BT3, BT4 and BT5).

All populations were identified as the Mediterranean species MED (Q1 mitotype) by sequencing part of their COI mtDNA gene and applying a diagnostic PCR to 50 females of each strain [87].

In all experiments, the laboratory MED strain S-GR6 susceptible to neonicotinoids was used as reference strain. It was collected in 2006 in Crete and has been since maintained in the lab without any insecticide treatment.

### Bioassays and selections

The selected strains were established by > 500 field collected individuals and were reared on cotton plants in standard insectary conditions (23–26 °C, 14:10 h Light:Dark, 70 % relative humidity). Selections were performed by exposing adult females and males for 72 h to a lethal concentration of commercial formulations of imidacloprid or acetamiprid. Survivors were transferred onto untreated cotton plants and allowed to lay eggs to start a new generation. The concentration of insecticide was adjusted at each generation (200 to 1200 μg/L imidacloprid, 100 to 1200 μg/L acetamiprid) in order to reach 60–80 % mortality after 72 h exposure. Surviving adults were transferred onto untreated cotton plants to obtain the next generation. In order to limit bottleneck effects, each generation was set off with > 300 individuals.

Full dose bioassays on cotton leaf discs were performed with female whiteflies as previously described [88, 89]. The insecticides used were: the neonicotinoids imidacloprid 200 g litre^−1^ SL (Confidor, Bayer AG, Leverkusen Germany), acetamiprid 20 SG (Profil, Nippon CO LYD, Japan) and thiacloprid 240 g litre^−1^ SC (CaLypso, Bayer AG, Leverkusen Germany). Final mortality was assessed after 72 h. Lethal concentrations corresponding to 50 % mortality (LC_50_) and their 95 % confident intervals (CI_95%_) were then calculated with a probit approach for each strain using PoloPC (LeOra Software, Berkeley, CA). Resistance ratios (RR_50_ based on LC_50_ values) were calculated by comparison to the susceptible strain.

Bioassays after exposure to piperonyl butoxide (PB, Sigma, UK), an inhibitor of the P450 dependent monooxygenases, were used to determine P450 metabolism contribution to resistant phenotypes. Piperonyl butoxide was applied at 300 ppm via a tarsal contact method 4 h prior to the insecticide application as previously described [69].

### RNA isolation, library construction and sequencing

In total, four different strains -all of MED species originated from Crete- were used in the RNA-Seq experiments: 1) the neonicotinoid susceptible strain S_GR6 was used as a reference strain 2) the resistant strain R_GR9_IS maintained under Imidacloprid Selection 3) the resistant strain R_GR4_AS maintained under Acetamiprid Selection, and 4) its corresponding Parental strain R_GR4_AP issued from the same field collected population (R_GR_4) which has been maintained in the laboratory without any selection pressure. Strains R_GR9_IS and R_GR4_AS were used as they exhibited the higher resistance levels against neonicotinoids.

Three biological replicates were included per strain in order to statistically validate the pair wise comparisons of transcript expression between the resistant and susceptible strains. For each biological replicate, total RNA was prepared by grinding approximately 300 frozen adults 1–2 days old, including males and females, using the Qiagen RNeasy mini kit according to the manufacturer’s protocol. Additionally, column digestion with DNAseI (Qiagen) was applied for the removal of genomic DNA contaminations. RNA concentration as well as the 260/280 and the 260/230 ratios were estimated using a Nanodrop spectrophotometer after which RNA samples were subjected to quality check with an Agilent Biolanalyzer, taking into account that in insects (and other taxa) the electrophoresis profile of rRNA significantly differs from the standard benchmark [90].

RNA samples were sent to Macrogen (Korea) for mRNA paired-end library construction with the Illunina TruSeqTM RNA Sample Preparation Kits v2 following the manufacturer protocol (Poly-A mRNA isolation with oligo-dT beads, mRNA fragmentation then followed by transcription into first-strand cDNA using reverse transcriptase and random hexamer primers) and sequencing on Illumina HiSeq 2000 platform. Barcoded libraries were prepared in a way that six samples could be run per lane. Each library was sequenced with the paired-end method for a read length of 100 base pairs.

### Read pre-processing

Read quality was assessed with FastQC [91] and PrinSeq [92]. For each sample, the raw reads were cleaned by removing adaptor sequences in two steps. First, using Scythe [93] to identify 3′-end adapters which often include poor quality bases. Then, SeqPrep [94] was used for both 5′ and 3′ adaptor removal. Low quality nucleotide reads trimming (Phred quality threshold of 25 and minimum reads length of 40 nt) was performed with Sickle, a sliding-window, adaptive, quality-based trimming tool for FastQ files (available at [95]). Finally, low complexity sequences (threshold entropy value of 60) and poly A/T 5′ tail (minimum of 5 A/T) trimming was performed using PrinSeq. To estimate ribosomal and mitochondrial RNA and remove the contaminant rRNAs, NCBI rRNA from Aleyrodidae, the SILVA rRNA database version 111 [96] and NCBI *Bemisia tabaci* mitochondrial genome were used. The reads from the fastq file were firstly collapsed (fastx_collapser from the fastx toolkit [97] to remove exact duplicate reads thus reducing the sequence number and a fasta file was produced. Then, ribokliper [98] (BLAT as aligner) was used to identify contaminant RNAs. At the end, the reads of each pair of all the libraries were re-combined and the remaining unpaired reads (singletons) were merged in a separate file using custom Perl scripts.

### Assembly

A preliminary assembly study was conducted in a subset of the dataset with SOAPdenovo-Trans [99] (kmers (17–77, steps of 2) optimal kmer 37, N50 = 500, average contig length = 293 and 214,371 contigs), Oases [100] (kmers (17–77, steps of 2) optimal kmer 35, N50 = 150, average contig length 136 and 668,034 contigs) and Trinity [101] (fixed kmers 25, N50 = 1961, average contig length 1032 and 107,274 contigs).

Based on the results of the preliminary assemblies, we selected to conduct our final de novo assembly using Trinity package. To increase transcriptome coverage and to facilitate downstream analyses (differential gene expression), *de novo* assembly was performed on a pool of the pre-processed reads (singleton and re-paired) from all the 12 samples. A single reference transcriptome was generated using Trinity package (release: 2013-02-25 with default parameters, kmers-25 and the minimum contig size fixed to 200 bp).

To assess the assembled transcripts quality, we followed four procedures: a) we pooled all the reads and mapped them to the assembly using Bowtie2 [102] and Samtools [103] to estimate the percentage of mapped reads, b) the quality of the assembled data was assessed by examining the similarity between the Trinity assembly and nr NCBI protein database using blastx search, c) the completeness of the produced contigs was estimated using a dataset of 129 complete mRNA of *B. tabaci* obtained from NCBI with blastn search, and d) with CEGMA (Core Eukaryotic Genes Mapping Approach) [104] we determined how many of 248 highly conserved eukaryotic genes were present in the *B. tabaci* transcriptome. In addition, based on BLAST NCBI taxonomy database we identified and removed 3, 467 host plant and fungus contaminant contigs (representing 2552 unigenes), as well as their respective mapped reads, from further analysis. No filtering against low-abundance contigs was performed in order to maximize the detection of low expressed transcripts. All the BLAST (http://www.ncbi.nlm.nih.gov/pubmed/2231712) searches were performed using NOBLAST program [105] (http://sourceforge.net/projects/noblast/).

### Homology searches and sequencing annotation

For sequence annotation of the assembled transcripts, first, a blastx similarity search against the NCBI protein database *nr* (*e*-value threshold 10^−6^; keeping the top 20 hits) was performed. Sequences that did not receive any hit were then searched via blastn against NCBI nucleotide database *nt* (*e*-value threshold 10^−10^; keeping the top 20 hits) using the parallel version of NOBLAST. For gene ontology (GO) mapping, Blast2GO [106] was used locally to recover all the GO terms associated to the hits obtained by the blast search. After the mapping step, results were subjected to GO annotation, a process of selecting GO terms from the GO pool and assigning them to the query sequences.

The sequences were further annotated using InterPro [107]. A local InterProScan (version 5, InterPro release 42.0) [108] was run in parallel (splitting the query set) on the longest open reading frame (ORF) of the contigs using a custom pair script based on the EMBOSS program “getorf” [109]. GO terms corresponding to these InterPro domains, were merged with the already existent GO terms derived from the blastx against *nr*.

GO terms were modulated using the annotation augmentation tool ANNEX [110] followed by GOSlim [111]. Enzyme classification (EC) codes were obtained through the direct mapping of GO terms to the corresponding enzyme codes. Sequences having EC numbers were further characterized by Kyoto Encyclopedia of Genes and Genomes (KEGG) [112] metabolic pathway annotations using custom perl/R scripts.

### P450s and nAChR similarity searches and phylogenetic inferences

For genes of particular interest expected to be involved in neonicotinoid resistance such as P450s and nAChR, similarity search and phylogenetic reconstructions were performed using known orthologs from other insect species.

*B. tabaci* contigs (>200 bp) encoding P450s and nAChRs were identified using BLAST, Blast2Go and interpro as described above. In addition, P450s and nAChRs were further identified using tblastn search (*e*-value cutoff <10^−10^) in protein sequences of other insect species from [33, 38, 113, 114] or downloaded from GenBank/ENSEMBL used as query against the *B. tabaci* assembly transcriptome. Contigs were translated and searched by Blastp against themselves. Results showing more than 99 % similarity were considered as allelic variants. Specifically for nAChR subunits, which displayed high expression variation (see Results and Discussion for details), the nucleotide sequence length of contigs included in phylogenetic analysis ranged from 346 bp (α6 subunit) to 5,473 bp (α4 subunit), with an average of 1,789 bp.

MAFFT-7.050 [115] was used to perform multiple sequence alignment of *B. tabaci* MED P450 protein sequences with a representative dataset of their counterparts in other species (Additional file 15: Figure S7).

Only protein sequences showing no misalignment were used in the final dataset for phylogenetic analysis.

Model selection was performed with ProtTest-3 [116] and the optimum model for phylogenetic analysis was selected according to Akaike information criterion (P450s: LG + G + F, nAChR: WAG + G + F). A maximum likelihood analysis was performed using RaxML v8 [117] and a bootstrap analysis with 1000 pseudoreplicates was performed to evaluate the branch strength of each tree. The resulting tree was midpoint rooted and edited with FigTree-1.4.1 software [118].

### Detection of polymorphism in the nAChR

Detection of single nucleotide polymorphisms (SNPs) was performed for the nAChR genes. Among all detected polymorphism variations, only SNP substitutions were considered for differential polymorphism analyses. Alignment files generated by Bowtie2 were used for SNP detection using SAMtools and Varscan [119] with the following parameters: map quality > 10, PHRED quality score > 25, coverage > 10 reads, *P* value threshold for calling variant = 0.01 and minimum supporting reads at a position to call variant = 4.

SNP allele frequencies were then computed between each insecticide resistant strain and the susceptible strain. Allele frequencies were considered as differential between an insecticide-selected strain and the susceptible strain (differential SNPs) if the total read coverage at SNP position between both strains was ≥ 50 and difference in allele frequency between strains  > 50 % [22].

SNPs were categorized to synonymous, non-synonymous and those that belong to the transcripts UTRs using a custom perl script. Non-synonymous SNPs were manually checked.

### Differential gene expression analysis

Differential expression (DE) analysis was performed at the “gene” (unigene) level by pair wise comparisons between strains. For the quantification, the paired reads of each sample were mapped to the transcriptome assembly with Bowtie2 and abundance was estimated with RSEM v. 1.2.4, as implemented in the trinity script *run_RSEM_align_n_estimate.pl*. The estimated expected counts for each sample (the sum of counts in each row >10) were extracted and used for the analysis of differential expression conducted in EdgeR-3.8.0 bioconductor package [120] using EdgeR-robust method to dampen the effect of outlier genes [121] with logFC > 1 and FDR < 0.05.

### Real time polymerase chain reaction

The expression levels of seven P450 genes found to be differentially expressed by the RNA-seq approach were validated using quantitative reverse transcription PCR (qRT-PCR). Primers were designed using the Oligo7 Primer Analysis Software (Molecular Biology Insights, Cascade, CO, USA), based on cDNA sequences retrieved from RNA-seq and are listed in Additional file 16: Table S9. The qRT-PCRs were performed using as template the same RNA used for the library construction. An aliquot of 2.8 μg total RNA from each of the three biological replicates, for each strain, served as a template for cDNA synthesis with Superscript III (Invitrogen, Carlsbad, CA, USA) using oligo-dT_20_, according to the manufacturer’s instructions. The resulting cDNAs were diluted 20 times in ultra-high quality water for qRT-PCR reactions using a Mini Opticon System (Biorad, Hercules, CA, USA). PCR reactions of 25 μl contained Fast Start SYBR Green Master Mix (Kapa-Biosystems), 0.12 μM of each primer (Additional file 16: Table S9) and 5 μl of diluted cDNA. Melt curve analysis was performed to test the specificity of amplicons. A serial dilution of cDNA was used to generate standard curves for each gene in order to assess PCR efficiency and quantitative differences among samples. The fold-change of each target gene, normalized to the 60S ribosomal protein L29 (RPL29), and relative to the susceptible reference strain S-GR6, was calculated according to the 2^-ΔΔCT^ method incorporating PCR efficiency [122].

All the computations were performed at the HCMR-Crete, high-performance computing bioinformatics platform. All the custom Perl, R and bash scripts are available from the authors upon request.

## Additional files

Additional file 1: Table S1. Statistics summary of library reads. (DOCX 14 kb) Additional file 2: Table S2. Assembly statistics summary. (DOCX 12 kb) Additional file 3: Figure S1. GO analysis by ontology category (level 2). A. Molecular function (MF); B. Biological process (BP); C. Cellular component (CC). (A)The majority of the MF are involved in “catalytic activity” (41.25 %) and “binding” (39.93 %), followed by “transporter activity” (6.9 %). (B)The largest subcategories found in the BP group were “metabolic process” (17.7 %) and “cellular process” (17.7 %). The largest subcategory found in CC group was “cell” which comprised 43.4 % of the contigs followed by “organelle” (24.12 %). (PDF 50 kb) Additional file 4: Table S3. Summary of KEGG pathway mapping of *B. tabaci* contigs. (XLSX 13 kb) Additional file 5: Figure S2. Amino acid alignment of nAChR subunits from *B. tabaci* transcriptome with available protein sequences from 7 insect species. List of species in the alignment: *D. Melanogaster* (Dmel), *A. mellifera* (Amel), *T. castaneum* (Tcas), *A. gambiae* (Agamb), *B. mori* (Bmori), *A. pisum* (Apisum), *B. tabaci* (Btab). (Examination of this file requires the BioEdit program (http://www.mbio.ncsu.edu/bioedit/bioedit.html)). (ALI 135 kb) Additional file 6: Figure S3. Amino acid alignment of *B. tabaci* nAChR subunits. Protein sequence alignment of *B. tabaci* nAChR subunits with D_a1 subunit of *D. melanogaster*. The numbers on the right indicate the amino acid position. The N-terminal signal peptides, the N-terminal region encompassing the six conserved domains loops (A-F) and the transmebrane domains were predicted by sequence similarity and using the SignalP4.1 [123] and TMHMM softwares [124]. Predicted N-terminal signal peptide leader sequences are shaded in yellow while the six ligand-binding loops (D, A, E, B, F, C) and the four transmembrane domains (TM1-4) are shaded in grey and green respectively. Alignment of predicted protein sequences (aa sequence average length for all subunits except α6 = 457; for α6 = 115) shows that all subunits possess two Cys separated by 13 residues, typical of LGIC subunits, as well as six conserved Loops (Loops A-F) located at the N-terminal domain. All subunits carry a predicted signal peptide at the N-terminus and four transmembrane motifs (TM1–TM4) at the C-terminal domain, which are involved in forming an ion channel. Loops between TM3 and TM4 were highly variable. The YxCC and GEK motifs are shaded in black. Amino acid mutations involved in imidacloprid resistance (R81T (*M. persicae* numbering), Y151S (*N. lugens* numbering)) are marked by inverted red triangle while non-synonymous SPNs found in *B. tabaci* strains are shaded in blue and marked with asterisk. (PDF 39 kb) Additional file 7: Figure S4. PCA plot. Overall similarity between samples using mapped reads on all contigs of the reference transcriptome (DESeq). The global gene expression pattern shows a clear separation between the susceptible and the three resistant strains. The higher correlation was observed among biological replicates of the same strain followed by the correlation between samples of the GR4_AP and the GR4_AS strains. The higher variance was found within strain R_GR4_AS: one biological replicate is closer to the parental strain R_GR4_AP while the two others are separated indicating signals of selection under insecticide pressure. (PDF 17 kb) Additional file 8: Table S4. Differentially expressed unigenes (LogFC > 1, FDR < 0.05), with LogCPM and TMM normalized FPKM for each library with best blast hit. (XLSX 1123 kb) Additional file 9: Figure S5. Heatmap spearman correlation matrix of significant DE (LogFC > 1 FDR < 0.05) unigenes based on their TMM-FPKM and GO enrichment analysis. The relative expression levels of each gene (row) in the susceptible and resistant samples (column) are shown. Up- and down-regulated genes are represented respectively by red and yellow. Clustering analysis was performed using Euclidean distance and dward algorithm with optimal leaf order (unigenes) or Spearman’s rank correlation (12 samples) using mean-centering transformation. The cluster was cut in three different parts using “cutree” and the unigenes of each sub cluster were recovered for GO enrichment analysis with Blast2GO for GO enrichment analysis using all DE as reference. Clustering of the samples grouped the neonicotinoid resistant samples together. Based on the transcription level of the 3,745 DE unigenes, three sub-clusters showing marked differences in normalized FPKM were identified. Assigning known transcripts to molecular functions revealed differences in the GO enrichment in the three clusters, displayed in the figure. The sub-clusters A1 and A2 group unigenes with lower expression profile in the susceptible strain and differ in expression profiles between the resistant strains. The sub-cluster A1 includes transcripts highly over-expressed in GR4-AS and GR4-AP and the more frequent GO terms included transferase activity (24 %), ATP binding (23 %) and heme binding (20 %). The sub-cluster A2 displayed the more visible and more abundant differences between the GR9-IS and the other resistant strains and revealed an enrichment in terms related to cytochrome P450s such as heme binding, iron ion binding and oxidoreductase activity (60 % of all overrepresented GO terms). Terms related to hydrolase activity were also enriched in sub-cluster A2. The cluster B represents unigenes mostly up regulated in the susceptible strain. The cluster B is the most diversified with 16 GO terms enriched, the majority of which were related with signal transduction. (PDF 261 kb) Additional file 10: Table S5. GO enrichment analysis of all strains vs full transcriptome. The file consists separate sheets of up and down regulated GO enrichment terms of each resistant strain (GR4-AP, GR4-AS, GR9-IS) vs susceptible strain S-GR6 and of GR4-AS vs GR4-AP against full transcriptome. (XLS 621 kb) Additional file 11: Table S6. List of DE detoxification unigenes over-expressed in the three resistant strains. (XLSX 27 kb) Additional file 12: Table S7. List of contigs encoding P450 genes in the *B. tabaci* transcriptome. (XLSX 48 kb) Additional file 13: Figure S6. Phylogenetic analysis of *B. tabaci* P450s. Maximum likelihood phylogenetic tree of *B. tabaci Med* (BtMed, indicated in red) and P450 protein sequences from 14 insect species. Sequences were aligned using mafft and a bootstrapped midpoint-rooted tree was constructed (1000 bootstraps). Bootstrap percentage is indicated for crucial branches. List of species in the figure: *D. melanogaster* (Dmel), *L. striatellus* (Lstri), *B. dorsalis* (Bdor), *D. citri* (Dcit), *H. zea* (Hzea), *D. punctata* (Dpun), *A. gossypii* (Agoss), *A. gambiae* (Agam), *M. Domestica* (Mdom), *P. polyxenes* (Ppol), *A. pisum* (Apisum), *A. melifera* (Amel), *M. persicae* (Mpersi), *B. tabaci* (Btab). For accession numbers see tree or Additional file 15: Figure S7. (PDF 1005 kb) Additional file 14: Table S8. Quantitative PCR analysis table of 7 P450s expression levels in five field populations of *Bemisia tabaci* and their resistance ratios to imidacloprid and acetamiprid, compared to the susceptible strain S-GR6. (DOCX 12 kb) Additional file 15: Figure S7. Amino acid sequences (fasta format) of P450s from *B. tabaci* transcriptome with available protein sequences from other insect species included in the phylogeny (Additional file 13: Figure S6). (FAS 223 kb) Additional file 16: Table S9. List of primer used in real-time quantitative PCR. (DOCX 12 kb)
