# Supplementary material for: Transcription analysis of neonicotinoid resistance in Mediterranean (MED) populations of B. tabaci reveal novel cytochrome P450s, but no nAChR mutations associated with the phenotype
Source: BMC Genomics. 2015 Nov 14;16:939. doi: 10.1186/s12864-015-2161-5 (PMC4647701; doi:10.1186/s12864-015-2161-5)
Supplement: Additional file 1: Table S1. — Statistics summary of library reads. (DOCX 14 kb) [file 12864_2015_2161_MOESM1_ESM.docx]

| **Additional file 1_ Table S1: Statistics summary of library reads.** | | | | | | | | | |
| --- | --- | --- | --- | --- | --- | --- | --- | --- | --- |
| **Libraries** | **Raw reads count** | **Cleaned Reads (paired end)** | **Len mean** | **Qual mean** | **% GC** | **Total bases** | **% rRNA** | **% Hemiptera rRNA** | **% Mitochondrial** |
| **R_GR4_AP_1** | 60389544 | 28413626 | 99,03 | 38,32 | 43,62 | 5627841431 | 5,203183149 | 0,712855163 | 4,123412478 |
| **R_GR4_AP_2** | 64725690 | 29806616 | 98,1 | 37,91 | 44,37 | 5847803878 | 4,660260662 | 0,835026022 | 3,540972246 |
| **R_GR4_AP_3** | 64397944 | 29678072 | 98,1 | 37,91 | 44,4 | 5822958289 | 5,638570457 | 1,329490002 | 3,943628818 |
| **R_GR4_AS_1** | 68882128 | 32506555 | 99,01 | 38,32 | 44,54 | 6436967467 | 4,983348743 | 0,657224366 | 3,97896978 |
| **R_GR4_AS_2** | 67084054 | 31249603 | 98,77 | 38,25 | 45,61 | 6173076368 | 5,89974855 | 2,394331218 | 2,729497076 |
| **R_GR4_AS_3** | 69245788 | 31878250 | 98,06 | 37,88 | 44,42 | 6251676888 | 5,237533428 | 1,016972387 | 3,874613569 |
| **R_GR9_IS_1** | 68925530 | 32470637 | 99,02 | 38,32 | 44,15 | 6430704255 | 5,036944609 | 1,079427546 | 3,627665204 |
| **R_GR9_IS_2** | 63729158 | 29308255 | 98,09 | 37,91 | 44,42 | 5749738124 | 5,01851782 | 0,99419771 | 3,731685834 |
| **R_GR9_IS_3** | 61899622 | 28457495 | 98,17 | 37,92 | 42,77 | 5587408098 | 7,331105566 | 1,27313033 | 5,533821582 |
| **S_GR6-1** | 66745448 | 31290104 | 98,99 | 38,31 | 43,82 | 6195121296 | 4,898542363 | 0,781208653 | 3,815183868 |
| **S_GR6-2** | 77634038 | 36604848 | 98,99 | 38,3 | 45,2 | 7246953959 | 3,803253055 | 0,581679782 | 2,993912173 |
| **S_GR6-3** | 59438436 | 27379267 | 98,17 | 37,91 | 43,72 | 5375392109 | 6,594139281 | 1,118207438 | 5,072422136 |
|  |  |  |  |  |  |  |  |  |  |
|  |  |  |  |  |  |  |  |  |  |
