# Supplementary material for: Transcription analysis of neonicotinoid resistance in Mediterranean (MED) populations of B. tabaci reveal novel cytochrome P450s, but no nAChR mutations associated with the phenotype
Source: BMC Genomics. 2015 Nov 14;16:939. doi: 10.1186/s12864-015-2161-5 (PMC4647701; doi:10.1186/s12864-015-2161-5)
Supplement: Additional file 2: Table S2. — Assembly statistics summary. (DOCX 12 kb) [file 12864_2015_2161_MOESM2_ESM.docx]

| **Additional file 2_ Table S2: Assembly statistics summary.** | | |
| --- | --- | --- |
| **Total nb seq:** | **170377** |  |
| Total length (bp): | 193485321 |  |
| **Average length (bp):** | **1136** | 180 amino acids |
| Lower quartile (Q1) (bp): | 271 |  |
| Median (Q2) (bp): | 443 |  |
| Upper quartlie (Q3) (bp): | 1228 |  |
| Min size (bp): | 201 |  |
| Max size (bp): | 33242 | 10391 amino acids |
| Total Ns: | 0 |  |
| Total %GC: | 40 |  |
|  |  |  |
| N25: | 4978 |  |
| **N50:** | **2681** | 446 amino acids |
| N75: | 1012 |  |
| N90: | 371 |  |
|  |  |  |
| **Total trinity components** | **113450** |  |
|  |  |  |
| contaminations |  |  |
| comp removed | 2552 |  |
| contigs removed | 3467 |  |
|  |  |  |
