# Supplementary figures and images for: Transcription analysis of neonicotinoid resistance in Mediterranean (MED) populations of B. tabaci reveal novel cytochrome P450s, but no nAChR mutations associated with the phenotype
Source: BMC Genomics. 2015 Nov 14;16:939. doi: 10.1186/s12864-015-2161-5 (PMC4647701; doi:10.1186/s12864-015-2161-5)

**A**

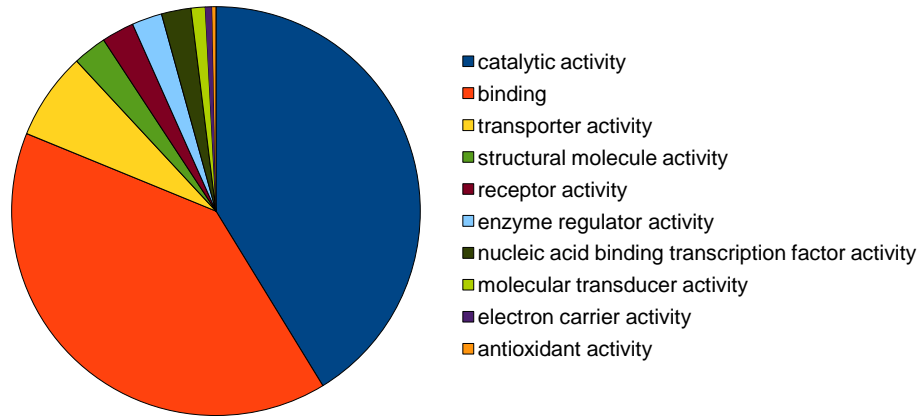

**B**

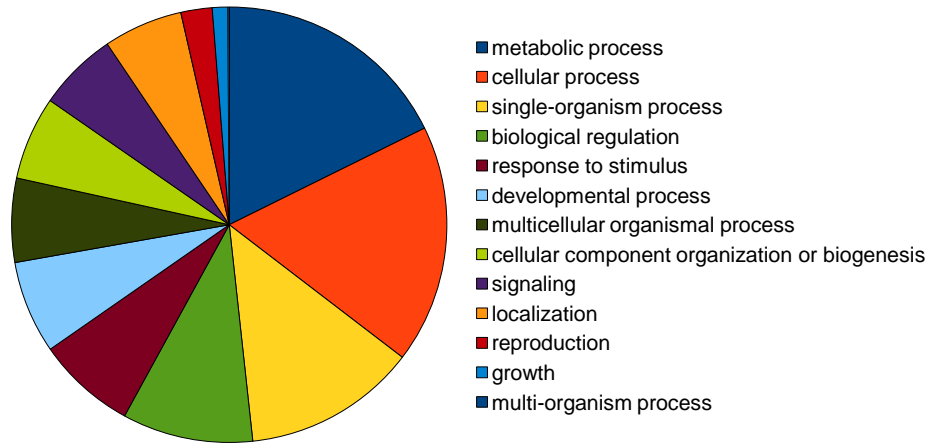

**C**

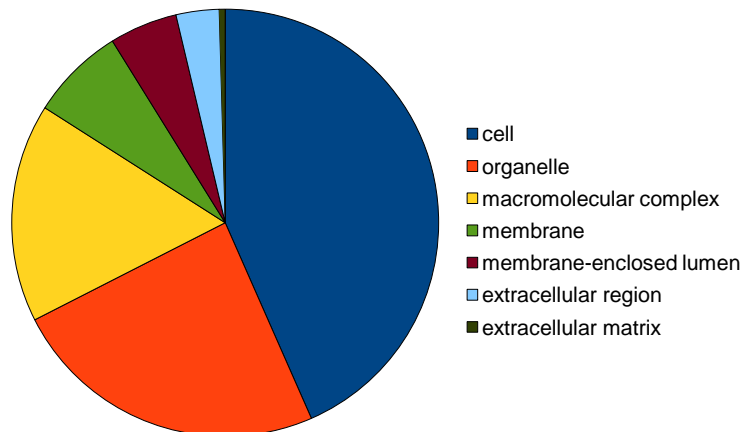

Supplement: Additional file 3: Figure S1. — GO analysis by ontology category (level 2). A. Molecular function (MF); B. Biological process (BP); C. Cellular component (CC). (A)The majority of the MF are involved in “catalytic activity” (41.25 %) and “binding” (39.93 %), followed by “transporter activity” (6.9 %). (B)The largest subcategories found in the BP group were “metabolic process” (17.7 %) and “cellular process” (17.7 %). The largest subcategory found in CC group was “cell” which comprised 43.4 % of the contigs followed by “organelle” (24.12 %). (PDF 50 kb) [file 12864_2015_2161_MOESM3_ESM.pdf]

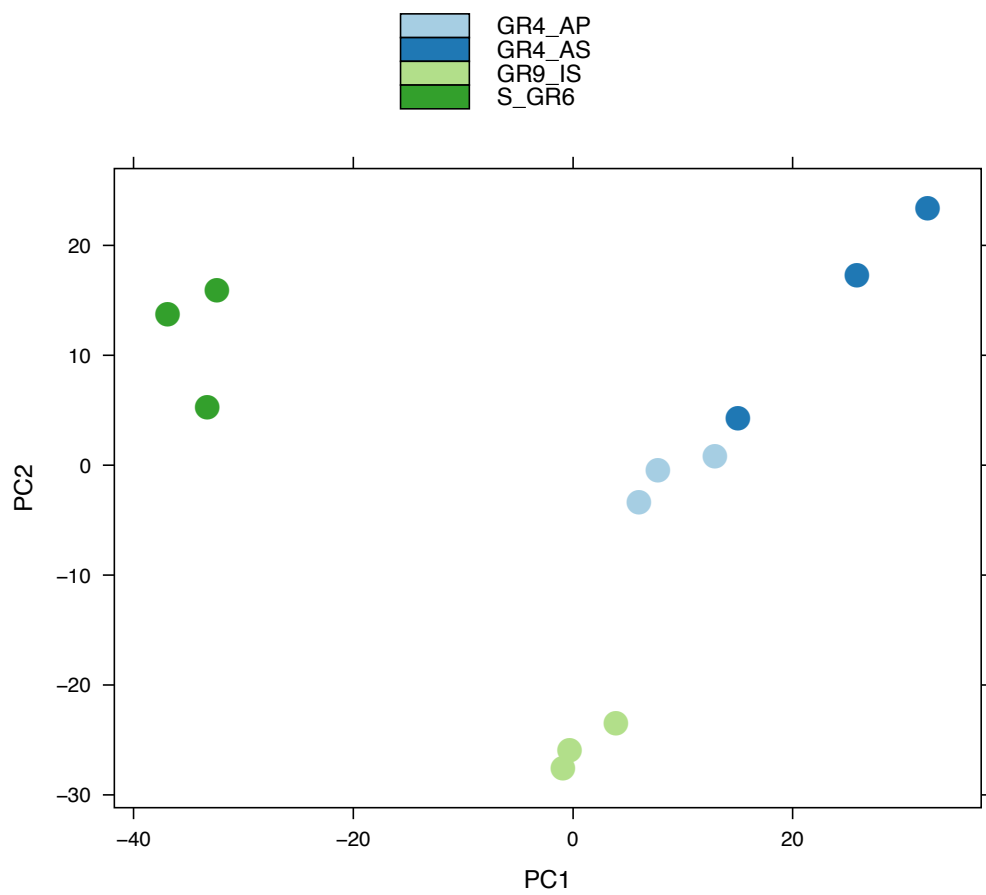

Supplement: Additional file 7: Figure S4. — PCA plot. Overall similarity between samples using mapped reads on all contigs of the reference transcriptome (DESeq). The global gene expression pattern shows a clear separation between the susceptible and the three resistant strains. The higher correlation was observed among biological replicates of the same strain followed by the correlation between samples of the GR4_AP and the GR4_AS strains. The higher variance was found within strain R_GR4_AS: one biological replicate is closer to the parental strain R_GR4_AP while the two others are separated indicating signals of selection under insecticide pressure. (PDF 17 kb) [file 12864_2015_2161_MOESM7_ESM.pdf]

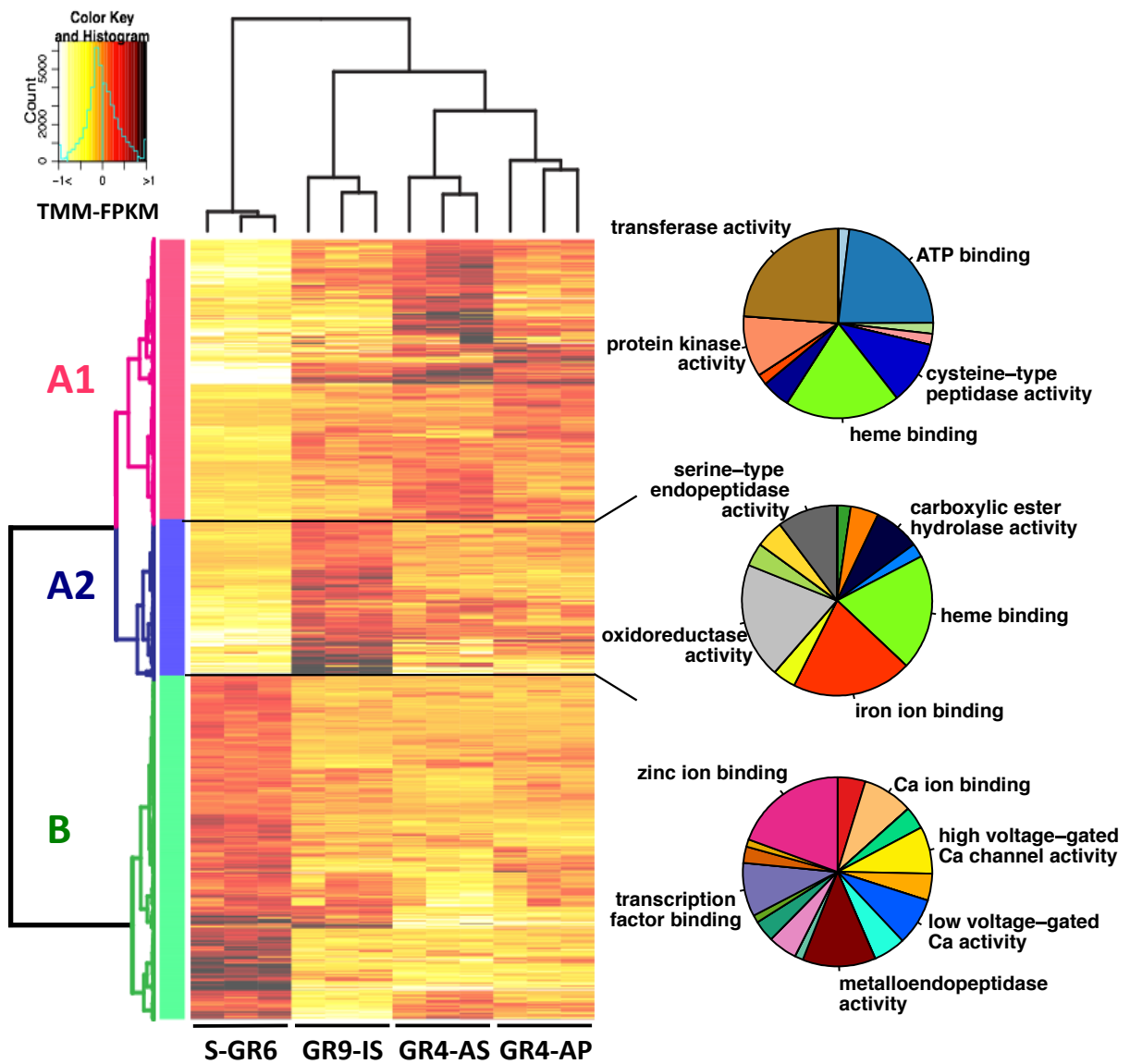

Supplement: Additional file 9: Figure S5. — Heatmap spearman correlation matrix of significant DE (LogFC > 1 FDR < 0.05) unigenes based on their TMM-FPKM and GO enrichment analysis. The relative expression levels of each gene (row) in the susceptible and resistant samples (column) are shown. Up- and down-regulated genes are represented respectively by red and yellow. Clustering analysis was performed using Euclidean distance and dward algorithm with optimal leaf order (unigenes) or Spearman’s rank correlation (12 samples) using mean-centering transformation. The cluster was cut in three different parts using “cutree” and the unigenes of each sub cluster were recovered for GO enrichment analysis with Blast2GO for GO enrichment analysis using all DE as reference. Clustering of the samples grouped the neonicotinoid resistant samples together. Based on the transcription level of the 3,745 DE unigenes, three sub-clusters showing marked differences in normalized FPKM were identified. Assigning known transcripts to molecular functions revealed differences in the GO enrichment in the three clusters, displayed in the figure. The sub-clusters A1 and A2 group unigenes with lower expression profile in the susceptible strain and differ in expression profiles between the resistant strains. The sub-cluster A1 includes transcripts highly over-expressed in GR4-AS and GR4-AP and the more frequent GO terms included transferase activity (24 %), ATP binding (23 %) and heme binding (20 %). The sub-cluster A2 displayed the more visible and more abundant differences between the GR9-IS and the other resistant strains and revealed an enrichment in terms related to cytochrome P450s such as heme binding, iron ion binding and oxidoreductase activity (60 % of all overrepresented GO terms). Terms related to hydrolase activity were also enriched in sub-cluster A2. The cluster B represents unigenes mostly up regulated in the susceptible strain. The cluster B is the most diversified with 16 GO terms enriched, the majority of which were relate [file 12864_2015_2161_MOESM9_ESM.pdf]

- CYP2
- CYP3
- CYP4
- Mitochondrial

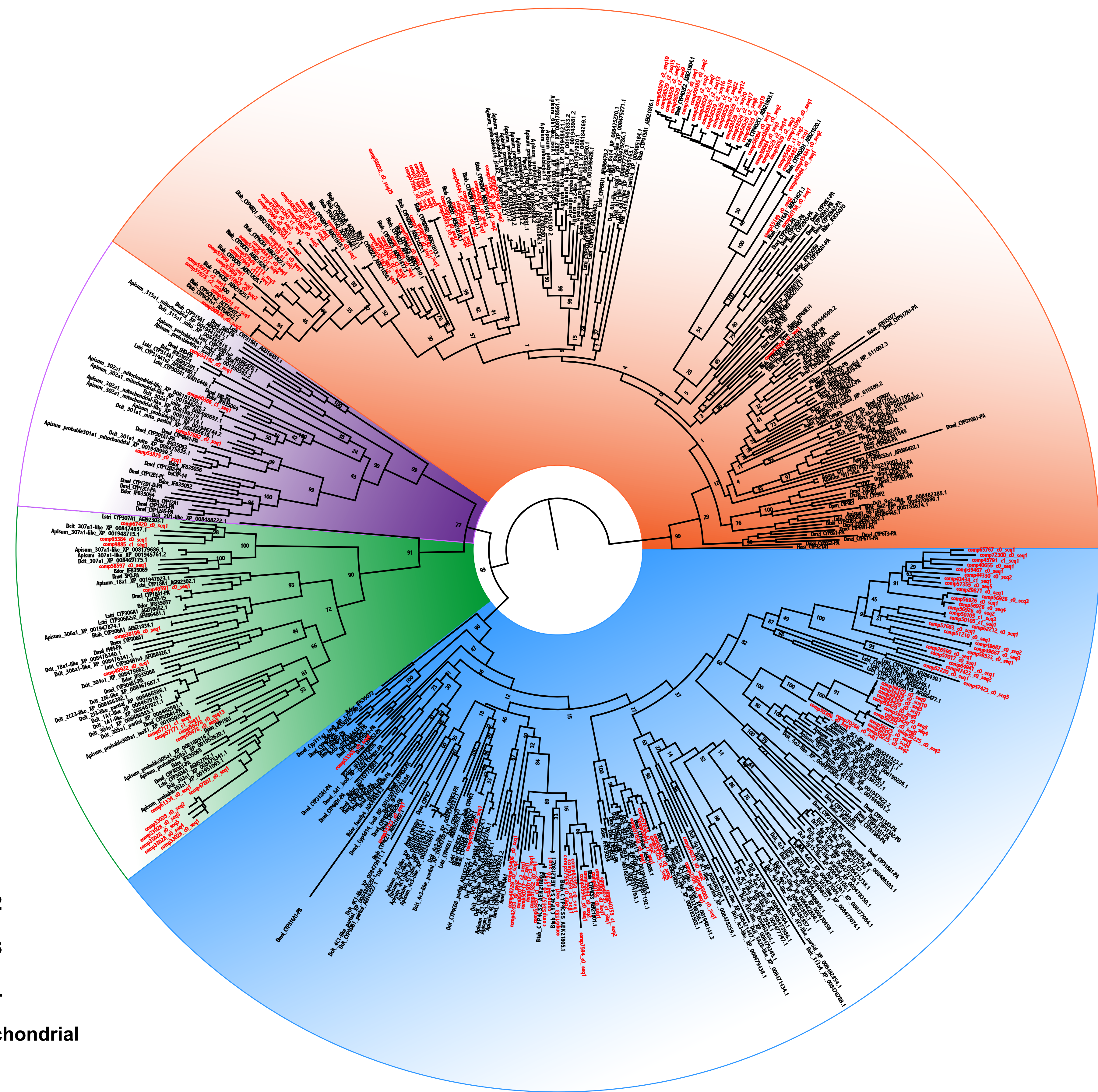

Supplement: Additional file 13: Figure S6. — Phylogenetic analysis of B. tabaci P450s. Maximum likelihood phylogenetic tree of B. tabaci Med (BtMed, indicated in red) and P450 protein sequences from 14 insect species. Sequences were aligned using mafft and a bootstrapped midpoint-rooted tree was constructed (1000 bootstraps). Bootstrap percentage is indicated for crucial branches. List of species in the figure: D. melanogaster (Dmel), L. striatellus (Lstri), B. dorsalis (Bdor), D. citri (Dcit), H. zea (Hzea), D. punctata (Dpun), A. gossypii (Agoss), A. gambiae (Agam), M. Domestica (Mdom), P. polyxenes (Ppol), A. pisum (Apisum), A. melifera (Amel), M. persicae (Mpersi), B. tabaci (Btab). For accession numbers see tree or Additional file 15: Figure S7. (PDF 1005 kb) [file 12864_2015_2161_MOESM13_ESM.pdf]
