# Supplementary material for: Transcription analysis of neonicotinoid resistance in Mediterranean (MED) populations of B. tabaci reveal novel cytochrome P450s, but no nAChR mutations associated with the phenotype
Source: BMC Genomics. 2015 Nov 14;16:939. doi: 10.1186/s12864-015-2161-5 (PMC4647701; doi:10.1186/s12864-015-2161-5)
Supplement: Additional file 6: Figure S3. — Amino acid alignment of B. tabaci nAChR subunits. Protein sequence alignment of B. tabaci nAChR subunits with D_a1 subunit of D. melanogaster. The numbers on the right indicate the amino acid position. The N-terminal signal peptides, the N-terminal region encompassing the six conserved domains loops (A-F) and the transmebrane domains were predicted by sequence similarity and using the SignalP4.1 [123] and TMHMM softwares [124]. Predicted N-terminal signal peptide leader sequences are shaded in yellow while the six ligand-binding loops (D, A, E, B, F, C) and the four transmembrane domains (TM1-4) are shaded in grey and green respectively. Alignment of predicted protein sequences (aa sequence average length for all subunits except α6 = 457; for α6 = 115) shows that all subunits possess two Cys separated by 13 residues, typical of LGIC subunits, as well as six conserved Loops (Loops A-F) located at the N-terminal domain. All subunits carry a predicted signal peptide at the N-terminus and four transmembrane motifs (TM1–TM4) at the C-terminal domain, which are involved in forming an ion channel. Loops between TM3 and TM4 were highly variable. The YxCC and GEK motifs are shaded in black. Amino acid mutations involved in imidacloprid resistance (R81T (M. persicae numbering), Y151S (N. lugens numbering)) are marked by inverted red triangle while non-synonymous SPNs found in B. tabaci strains are shaded in blue and marked with asterisk. (PDF 39 kb) [file 12864_2015_2161_MOESM6_ESM.pdf]

```
                                    N-terminal signal peptide
                  12(F/S)
                   10        20        30        40        50        60        70        80        90       100
           ....|....|.*..|....|....|....|....|....|....|....|....|....|....|....|....|....|....|....|....|....|
D_a1       ---------------------------------------MGSVLFAAVFIALHFA-------TGGLANPDAKRLYDDLLSNYNRLIRPVGNNSDRLTVKM 54
Btab_a1    PDNLPGLITVNDGTNPKCRNGNGT---------------MLGVVRMERFVVLAMV-------AVVAGNPEAKRLYNDLLSNYNRLIRPVGNNTDRLTVKM 78
Btab_a2    ---VIVEVREGESAGAMCFR--------------------EWLFVFLLILVYAL-------TMARGNPDAKRLYDDLLSNYNRLIRPVSNNTNTVLVKL 69
Btab_a3    --SRDHDTKESRPTNHGNMR----------------------IIYWIIVVLIS---------DCVCNPDAKRLYDDLLSNYNKLVRPVVNVTDALTVKI 66
Btab_a4    -RETPSVLSASRPWRFADLWRYLCRGHVQKLLTHADAMCVVETVRAWLLSALVVH-------VAVAGNPDAKRLYDDLLSNYNKLVRPVVNTTDVLRVCI 92
Btab_a5    --STAMRLLSDNR-----------------------------IFALLCVHSLLS------FVSVICDGVEYALMRTLMENYDSSIRPAKNSTQSLKVEF 62
Btab_a6    ---------------------------------------------------------------------------------------------------- 1
Btab_a7    ---EPAVWRHAVVFNLDMVR----------------------IGVVLVLCLLWT------PQESSQGPHEKRLLHTLLDTYNTLERPVANESEPLQLSF 68
Btab_a8    --DLSKYTTVTFSYYWISLITS-----------------TMLFVFALILLKLSYHPENVNGLKQIEANPDAKRLYDDLLSNYNRLIRPVVNNTETLVVQL 81
Btab_b1    IAATKEDIFTPECTGVDRHDTHSLG---------------VTPSMKTSLVATLNLLMIG--LILPCLCSEDEERLVRDLFRGYNKLIRPVQNITEKVDVRF 84
Btab_b2    ISVCAWSYFIHFNMIAKRFLDLGMVVIIFLALLKCSSSLKGSKYFMLGQQLSTTARPGGDGVNRLWDPTWTDRLKTDLLKSYDKYSRPTEHQN--ITTVY 98
                               LpD                               LpA                         LpE
                             R81T                                         159(F/Y)
                  110       120       130       140       150       160       170       180       190       200
           ....|....|....|....|....|....|....|....|....|....|....|....|....|.*..|....|....|....|....|....|....|
D_a1       GLRLSQLIDVNLKNQIMTTNVWVEQEWNDYKLKWNPDDYGGVDTLHVPSEHIWHPDIVLYNNADGNY-EVTIMTKAILHHTGKVVWKPPAIYKSFCEIDV 153
Btab_a1    GLKLSQLIDVNLKSQIMTTNMWVEQEWNDYKLKWNPEEYGGVDTLHVPSEHIWLPDIVLYNNADGNY-EVTIMTKAIIHHTGKVVWKPPATYKSFCEIDV 177
Btab_a2    GLRLSQLIDLNLKDQILTTNVWLEHEWEDHKFRWDPAEYGGCKELYVPSEHIWLPDIVLYNNADGEY-VVTTMTKAVLHFDGKVVWTPPAIFKSSCEIDV 168
Btab_a3    KLKLSQLIDVNLKNQIMTTNLWVEQSWNDYKLKWDPKEYGGVEMLHVPSDHIWRPDIVLYNNADGNF-EVTLATKATLNYTGRVEWKPPAIYKSSCEIDV 165
Btab_a4    KLKLSQLIDVNLKNQIMTTNLWVEQSWYDYKLRWEPKEYGGVHMLHVPSDHIWRPDIVLYNNADGNF-EVTLATKATIYNQGLVEWKPPAIYKSSCEIDV 191
Btab_a5    GVSLHHIIDVDEKNQILTTNCWITQVWTDHHLLWNISDFDEITVLRIPYHKVWRPDIILYNNADSQYNSAVINTNVIVKHDGEVTWLSHGIYRSSCDIDV 162
Btab_a6    --------------------------------GWNSSDYGGVKDLRITPNRVWKPDILMYNSADEKF-DGTFHTNVLVRNNGTCLYIPPGIFKSTCKIDI 67
Btab_a7    GLTLMQIIDVDEKNQLLITNIWLKLEWNDVNLRWNPSEFGNVRDLRIPPHRLWKPDILMYNSADEGF-DGTYPTNVVVRSNGSCSYIPPGIFKSTCKIDI 167
Btab_a8    RLKMSQLLEVSLKSQVMTTNVWVEQKWNDYKLRWDPNEYGGVEMLYVPSEHIWLPDIVLYNNADGNY-EVTLMTKATLNYTGDVLWRPPAIYKSSCEMNV 180
Btab_b1    GLAFVQLINVNEKNQIMKSNVWLRLVWNDYQLRWDETDYGGIGVLRLPPDKVWKPDIVLFNNADGNY-EVRYKSNVLIYPHGEVLWIPPAIYQSSCTIDV 183
Btab_b2    LSLILLHVSLDYSKSEMALNCWLPMSWQDEKLKWNTSDYNGISRINIPQHEIWLPDILAYNSAHGNIIDYYGQTSTIVKSDGSVLWVPPSRFTVFCTLDL 198
                               LpB        LpB-C       LpF                      LpC                     TM1
                        Y151S                                                 YxCC
                  210       220       230       240       250       260       270       280       290       300
           ....|....|....|....|....|....|....|....|....|....|....|....|....|....|....|....|....|....|....|....|
D_a1       EYFPFDEQTCFMKFGSWTYDGYMVDLRHLKQTADSDNIEVGIDLQDYYISVEWDIMRVPAVRNEKFYSCCEEPYLDIVFNLTLRRKTLFYTVNLIIPCVG 253
Btab_a1    EYFPFDQQTCFMKFGSWSYDGYTVDLRHISQTPDSTEIEMGIDLQDYYLSVEWDVMRVPATRNEKFYSCCEEPYQDIMFNITLRRKTLFYTVNLIIPCIG 277
Btab_a2    RYFPFDQQTCFMKFGSWSYDGNQIDLKHINQKAGADMVEVGIDLREYYPSVEWDILGVPAERHEKYYPCCKEPYPDIFFNLTLRRKTLFYTVNLIVPCVG 268
Btab_a3    EYFPFDEQTCVMKFGSWTYDGFQVDLRHIDEVFGSNVVNIGVDLSEFYTSVEWDILEVPAVRNEKFYTCCDEPYLDITFNITMRRKTLFYTVNLIIPCMG 265
Btab_a4    EYFPFDEQTCVLKFGSWTYDGFMVDLRHMDEKVGSNIVEVGVDLSEFYMSVEWDILEVPAVRNEKFYTCCDEPYLDITFNITMRRKTLFYTVNIIIPCMG 291
Btab_a5    EYFPFDIQSCAMKWASWTYDGYMLDLVKQTEEG---------DVSNYQANGEFDLIGFDSIKNVITYSCCEEPYPDITYFIRLRRRPMFYVFNLILPCIL 253
Btab_a6    TWFPFDDQKCDMKFGSWTYDGFQLDLQLKSEEGG--------DLSDFIPNGEWYLL---------------------------------------------- 115
Btab_a7    TWFPFDDQRCEMKFGSWTYDGFQLDLQLQDEGGG--------DISSFITNGEWDLLGVPGKKNVIYYNCCPEPYIDITFVIIRRRTLYYFFNLIVPCVL 259
Btab_a8    LYFPFDEQSCLMKFGSWTYNGFQIDLKHMEQVYGSNLVNVGVDLSEFHLSVEWDILEVPARRNEEYYPCCTEPYSDITFNITMRRKTLFYTVNLIIPCVG 280
Btab_b1    TYFPFDQQTCIMKFGSWTFNGDQVSLALYNNKN-------FVDLSDYWKSGTWDIIEVPAYLN--TYPGVPT-ETDITFYIIIRRKTLFYTVNLILPTVL 273
Btab_b2    TYWPYDQQTCQLRLGSWSYDGNSIDVQLDKAKS---------SIEDLEVS-EWKVIDVSQKRFVQYYHHCPEPYVEIIFSYTVKKESPAFFYTIIFPAIV 288
             TM1                   TM2                                TM3
                      GEK
                  310       320       330       340       350       360       370       380       390       400
           ....|....|....|....|....|....|....|....|....|....|....|....|....|....|....|....|....|....|....|....|
D_a1       ISFLSVLVFYLPSDSGEKISLCISILLSLTVFFLLLAEIIPPTSLTVPLLGKYLLFTMMLVTLSVVVTIAVLNVNFRSPVTHRMAPWVQRLFIQILPKLL 353
Btab_a1    LSFLSVLVFYLPSDSGEKVSLCISILLSLTVFFLLLAEIIPPTSLTVPLLGKYLLFTMVLVTLSVVVTIIVLNVNFRSPVTHKMTPFWYKVFIQVLPKVL 377
Btab_a2    ISYLSVLVFYLPADSREKVVLCITILLSQTMFFLLISEIIPSTSLALPLLGKYLLFTLVLIGLCVVITIGVLNVHYRKPSTHKMAPWVRKLFIRRLPRLL 368
Btab_a3    ISFLTVLVFYLPSDSGEKVSLSISILLSLTVFFLLLAEIIPPTSLVVPLLGKFVLFTMILDTFSICVTVVVLNVHFRSPQTHTMAPWVKRVFIHILPKLL 365
Btab_a4    ISFLTVLTFYLPSDSGEKVTLSISILISLHVFFLLVVEIIPPTSLVIPLLGKYLIFAMILVSISICVTVVVLNVHFRSPQTHKMAPWVKRVFIHILPRLL 391
Btab_a5    INCIALLVFYVPSESGEKVTLGISALLSMTVFLMTIRETLPPT-EKTPLISMYYGVSICLVTLASGLSVVTLNIYHRGVRGTKVPGIIKATMLGKLARCL 352
Btab_a6    ---------------------------------------------------------------------------------------------------- 115
Btab_a7    IASMAVLGFTLPPDSGEKLSLGVTILLSLTVFLNMVAETMPATSDAVPLLGTYFNCIMFMVASSVVSTILILNYHHRNADTHEMSPWIKLLFLKWFPCIL 359
Btab_a8    ITFLTVLVFYLPSDSGEKVSLCSSILVSLTVFFLLLAEIIPPTSLAVPLLGKYLLFTMTLVTLSICVTVCVLNVHFRSPSTHKMSPWVKTVFLHFMPRLL 380
Btab_b1    ISFLCVLVFYLPAEAGEKVTLGISILLSLVVFLLLVSKILPPTSLVLPLIAKYLLFTFIMNTVSILVTVIIINWNFRGPRTHRMPVWIRSVFLYYLPIIL 373
Btab_b2    TMMLTMSAFWLPPESMERFILNG-ICAVIAVLFLIYFSIKLPIMVHTPLIVVFYSTVLGLMGVSLGIFIAMDNLRKQESHPKPISRLLSSIADFRLGQNF 387
```

```
                                             400(D/E)                  426(N/T)                    448(P/A)
                   410       420       430       440       450       460       470       480       490       500
            ....|....|....|....|....|....|....|.*..|....|....|....|....|..*.|....|....|....|....|....|*...|....|
D_a1        CIERPKKEEPEEDQPPE-VLTDVYHLPPDVDKFVNYD-SKRFSGDYGIPALPASHRFDLAAAGG-----ISAHCFAEPPLPSSLPLPGADDDLFSPSGLN 446
Btab_a1     MIERPNKDDSIDDDKPEGVLTGVFDADLGADKYLAYGGPEEYSPEYDIPEPPPS-RYEPNSAPAPVVVPLPLPCFEEPPPPS--PLPGPEDDLFSAPG-- 472
Btab_a2     LMRVPEQL------LIDMAAKKRLKNATSAKFNAALAASSLGSSTESLRGRHPYMPGGGPGCNGL----HANNGVTNRFNSLVGALSGSVEAMNNGHGHG 458
Btab_a3     VMRRPHYP--VDKR--GLAASCQRIMVRTCNGNVELRDSSLFPHHAAPPALPSELLNTPAQYVFQ----HSSRDTEPTIIGSSCEIHGAPPALSQLFPDT 457
Btab_a4     VMRRPQNPEGVPKINCGPITSTRATKGKEYEMYFYPRQTS-LEDLAKLPKLPKFNDDGFYDSKEQ----HSSGSLMDGPLSGSCQIHG--PITSSLMSEE 484
Btab_a5     FLQFEPRK---------------------KNSQEARRCGPKMQINYTKPDRWTTMNDEYRYNSPK----FIMRHSAQP---------------------- 405
Btab_a6     ---------------------------------------------------------------------------------------------------- 115
Btab_a7     RMSRPEPET------------PVSGSGESARKLQLRELELKERSSKSLLANVLDIDDDFRHAPIP---PPAPHGYLPRHGVGVGGHPH------------ 432
Btab_a8     MMRRPPYS----------------------PNDLYLENLSDASYVNDGDFRDSFSDGCPMEHKCS----PKENRNMYKSNDCNHFHHG------------ 442
Btab_b1     LMKRPKKT----------------------RLRWMMEMPSMTNVSSVPPHPHYGSPTELPKNLAQ----ASSKSKTEVMELSDFHHPN------------ 435
Btab_b2     LRPSPYST------------------------LLNSDERSKFGGIRTPSGGATGMPSMISNENLIG---------------------------------- 429
                                                                                            TM4

                   510       520       530       540       550       560       570       580       590       600
            ....|....|....|....|....|....|....|....|....|....|....|....|....|....|....|....|....|....|....|....|
D_a1        GD-------ISPGCCPAAAAAAAADLSPTFEKPY--AREMEKTIEGSRFIAQHVKNKDKFESVEEDWKYVAMVLDRMFLWIFAIACVVGTALIILQAPSL 537
Btab_a1     ------------GGCGNALCPRLAGRSPVFERP---LREIEKTIEDAKFIAQHVKNKDKFENVIEDWKYVAMVIDRLFLWLFLFACVIGTCLIILQAPSL 557
Btab_a2     -----------HGHGHPVVVAQDDNLSDAPQRKK-YPFELEKAIHNVKFIQHHILREDEFDAEDQDWGFVAMVLDRLFLWIFSITSIVGTFVILCEAPAL 546
Btab_a3     LGENGCGGVTPEPSPNENGSAVPTSSSHHLPQRWHHCPELHKAFDGVRYIADHTKKEEDSTKVKEDWKYVAMVLDRLFLWIFTLAVLVGSAGIILQAPTL 557
Btab_a4     LS--------LTPTPDDGKSPVLRNPAFSHSK---CPPIVHRSCFCMRFIAEHTRMIEESTKVKEDWKYVAMVLDRLFLWIFTLAVIAGTAGIILQAPTL 573
Btab_a5     ---------------AETTATSTGSRSFETQFMR----MLQRVNVTVEQNELRLLDQERREQTELEWKQVALVMDRLLLWIFIILTAISTTVILRGSPYG 486
Btab_a6     ---------------------------------------------------------------------------------------------------- 115
Btab_a7     --------------EDGHTGTLLHATSASCLGPH---RELALILKELRVITEKIRKEEEDAAVTNDWKFAAMVVDRLCLIIFTLFTVIATIAVLMSAPHI 515
Btab_a8     ------------AQAPDLENVIPRHLS----------DDLLAALEGVRFIAQHIKDADKDNEVIEDWKYVSMVMDRFFLWIFTVACIGGTCIIMFQAPSL 520
Btab_b1     -------------CKINRKASVERRESESSDSLL-LSPEASKATEAVEFIVEHLRNEDQYIQIREDWKYVAMVIDRLQLYIFFIVTTVGTVGILMDAPHI 521
Btab_b2     -------------------------------------------KKGKPQPYPLSADQTFLKPSSNEWRSLATLFHKISLVAHVLMYLIILIKCFY----- 481

                   610       620       630
            ....|....|....|....|....|....|....|
D_a1        HDQSQPIDILYSKIAKKKFELLKMGSENTL----- 567
Btab_a1     YDTTKPIDIVHSKVAKKKLRMMMMGPEED------ 586
Btab_a2     YDDTKPIDLQLSSVAQQQFLPDVTSDF-------- 573
Btab_a3     YDDRRPIDVQLSDIASTTSKPQVTTTTL------- 585
Btab_a4     YDDRIPIDKRFAEVAMSQPFNPCPPPK-------- 600
Btab_a5     P---------------------------------- 487
Btab_a6     ----------------------------------- 115
Btab_a7     IVN-------------------------------- 518
Btab_a8     YDERAPIDRQLSTFLYGENQMPIYSINRDKILDAL 555
Btab_b1     FEYVD--QDRIIEIYRGK----------------- 537
Btab_b2     ----------------------------------- 481
```
