# Supplementary material for: Transcription analysis of neonicotinoid resistance in Mediterranean (MED) populations of B. tabaci reveal novel cytochrome P450s, but no nAChR mutations associated with the phenotype
Source: BMC Genomics. 2015 Nov 14;16:939. doi: 10.1186/s12864-015-2161-5 (PMC4647701; doi:10.1186/s12864-015-2161-5)
Supplement: Additional file 14: Table S8. — Quantitative PCR analysis table of 7 P450s expression levels in five field populations of Bemisia tabaci and their resistance ratios to imidacloprid and acetamiprid, compared to the susceptible strain S-GR6. (DOCX 12 kb) [file 12864_2015_2161_MOESM14_ESM.docx]

| **Additional file 13_Table S8 : Quantitative PCR analysis of 7 P450s expression levels in five field populations of Bemisia tabaci and their Resistance ratios to imidacloprid and acetamiprid compared to susceptible strain S-GR6.** | | | | | | | | | |
| --- | --- | --- | --- | --- | --- | --- | --- | --- | --- |
|  |  |  |  |  |  |  |  |  |  |
|  | **Resistance Ratio vs S-GR6** | | **qPCR Fold regulation compared to susceptible strain S-GR6*** | | | | | | |
|  | **Imidacloprid** | **Acetamiprid** | **comp50040_c0** | **comp57969_c113** | **comp57969_c124** | **comp33028_c0** | **comp61334_c0** | **comp43065_c0** | **comp43434_c1** |
| BT1 | 6 | 2 | 7.01 | 1.16 | 0.88 | 0.28 | 18.38 | 2.04 | 1.13 |
| BT2 | 65 | 7.5 | 20.12 | 2.44 | 3.00 | 0.99 | 187.55 | 1.31 | 1.58 |
| BT3 | 19 | 26.5 | 4.76 | 3.07 | 5.42 | 0.30 | 22.58 | 0.69 | 1.02 |
| BT4 | 12 | 24.3 | 4.95 | 4.16 | 4.96 | 0.77 | 70.55 | 1.22 | 2.11 |
| BT5 | 13 | 22.5 | 9.38 | 2.37 | 3.10 | 1.10 | 64.60 | 1.31 | 1.51 |
|  |  |  |  |  |  |  |  |  |  |
| * One biological replicate /population was examined. | | | | |  |  |  |  |  |
