# Supplementary material for: Transcription analysis of neonicotinoid resistance in Mediterranean (MED) populations of B. tabaci reveal novel cytochrome P450s, but no nAChR mutations associated with the phenotype
Source: BMC Genomics. 2015 Nov 14;16:939. doi: 10.1186/s12864-015-2161-5 (PMC4647701; doi:10.1186/s12864-015-2161-5)
Supplement: Additional file 16: Table S9. — List of primer used in real-time quantitative PCR. (DOCX 12 kb) [file 12864_2015_2161_MOESM16_ESM.docx]

| **Additional file 14_Table S9: List of primer used in real-time quantitative PCR.** | | | |  |  |
| --- | --- | --- | --- | --- | --- |
| **Unigene** | **Annotation name** | **Primer name** | **Primer Sequence (5’ to 3’)** | | **Transcript length (bp)** |
|  |  |  | **Forward** | **Reverse** |  |
| comp50040_c0 | CYP6CM1vQ | Bt_qPCR_CYP6A14 | TCCAGCTTCTCTGGCAGATT | TATCACCGATGGCTCTCTCC | 208 |
| comp61334_c0 | CYP303A1-like | Bt_qPCR_ CYP303A1-like | CGCTCCGGTACAATGCTAATCG | CCCATACACCTGTGTTTACCGAAT | 167 |
| comp33028_c0 | CYP303A1 | Bt_qPCR_ CYP303A1 | CACGATGTGGATAGCCGTCT | TAATGCACACCCGATAAGAGG | 124 |
| comp57969_c113 | CYP6CX5 | Bt_qPCR_ CYP6CX5 | GACTTTCCAGCTGCTCAACCC | GTTCCCGCTGAGCTTGTCCA | 136 |
| comp57969_c124 | CYP6CX3 | Bt_qPCR_ CYP6CX3 | CGCATTCTTCCAGTTCCTCGAGA | GGCCATAGCATCCTTCGTGACC | 118 |
| comp43065_c0 | CYP4C1-like | Bt_qPCR_CYP4C1-like | AGGACCCGGAGAAATTCATACCAG | TGGCATACTTCAACCCCAAGCA | 120 |
| comp43434_c1 | CYP417B1 | Bt_qPCR_CYP417B1 | GAAGTACCAATTTTCAACGGACGAA | TGACTTCAAATCCTGCGGCTG | 101 |
|  |  | Bt_qPCR_ RPL29 | TCGGAAAATTACCGTGAGAA | GAACTTGTGATCTACTCCTCTCGTG | 144 |
